# Effects of Continuous Medicaid Coverage in 2020–2023 on Children's Health Insurance Coverage, Access to Care, Health Services Use by Type, and Health Status

**DOI:** 10.1111/1475-6773.70034

**Published:** 2025-08-31

**Authors:** Wei Lyu, George L. Wehby

**Affiliations:** ^1^ Department of Health Services Administration University of Alabama at Birmingham Birmingham Alabama USA; ^2^ Department of Health Management and Policy University of Iowa Iowa City Iowa USA; ^3^ Department of Economics University of Iowa Iowa City Iowa USA; ^4^ National Bureau of Economic Research Cambridge Massachusetts USA

**Keywords:** access to care, children, health insurance coverage, health status, insurance continuity, Medicaid

## Abstract

**Objective:**

To examine the effects of continuous Medicaid coverage in 2020–2023 under the Families First Coronavirus Response Act (FFCRA) on children's health insurance coverage, access to care, likelihood of using healthcare services by type, and health status.

**Study Setting and Design:**

A difference‐in‐differences event study compares outcomes pre and post FFCRA between states without pre‐FFCRA continuity provisions (treatment group) and those that required 12‐month continuous coverage (control group).

**Data Sources and Analytical Sample:**

The main sample includes 122,901–126,117 children (depending on outcome) aged 1–17 years with family income below 300% of federal poverty level from the 2016–2023 National Survey of Children's Health.

**Primary Findings:**

After FFCRA, public coverage increased in treatment states in 2020, 2021, and 2022 by 4.1 (95% CI: 0.004, 8.3), 4.7 (95% CI, 0.4, 9.0), and 5.4 (95% CI: 2.0, 8.7) percentage points, respectively, relative to control states. Privately purchased coverage declined in 2020 by 3.5 (95% CI: −5.3, −1.7) percentage points. The likelihood of having a usual place for sick care increased by 3.6 (95% CI: 0.5, 6.8) percentage points in 2021, and the likelihood of unmet care needs decreased by 1.7 (95% CI: −2.8, −0.7) and 2.4 (95% CI: −3.8, −1.0) percentage points in 2021 and 2022. The likelihood of excellent/very good health increased by 2.5 (95% CI: 0.4, 4.5), 3.8 (95% CI: 0.7, 6.8), and 2.7 (95% CI: 0.4, 5.0) percentage points in 2020, 2021, and 2023, respectively. There were no changes in the likelihood of medical, preventive, mental health, specialist, and emergency department visits and hospital admissions.

**Conclusions:**

Medicaid continuity under the FFCRA increased the children's public coverage rate. Despite potential switching from private coverage, there is evidence for reductions in unmet care needs and improved health status. Findings provide insights into potential effects of recent federal requirements that all states provide 12‐month Medicaid continuity for children.

**Summary**

- What is known on this topic
  ○ Coverage gaps among children have been associated with disruptions to sources of care, decreased use of preventive services, increased unmet care needs, emergency department visits, and potentially avoidable hospitalizations.
  ○ The Families First Coronavirus Response Act (FFCRA) prohibited states from disenrolling Medicaid beneficiaries through March 2023, which offered continuous coverage to three years.
  ○ There is emerging evidence showing that the FFCRA increased Medicaid enrollment and access to care for non‐elderly adults and more children enrolled in Medicaid.
- What this study adds
  ○ This study uses data from the 2016 to 2023 National Survey of Children's Health (NSCH) and a difference‐in‐differences event study design to study the effects of the FFCRA continuous Medicaid coverage provision on children's coverage, access, use of specific types of services, and health status.
  ○ There is an increase in children's public coverage, some of which may be switching from private coverage. There is also a reduction in unmet care needs and more reporting of excellent/very good health.
  ○ There are no apparent effects on the likelihood of medical, preventive, mental health, specialist, and emergency department visits and hospital admissions.

## 1 Introduction

Health insurance gaps among children in the United States are linked to significant disruptions in health care [1, 2, 3, 4, 5, 6, 7, 8, 9, 10]. Prior evidence indicates that coverage gaps reduce the likelihood of children having a usual source of care [1, 2, 3, 4], decrease the use of preventive services including well‐child visits and immunizations [1, 4, 5, 6, 7], increase the likelihood of unmet care needs [4, 8, 9], and increase emergency department visits [9] and potentially avoidable hospitalizations [10]. Before 2020, Medicaid coverage had to be renewed at least every 12 months or more often, potentially leading to insurance gaps for many children [11, 12]. In 2019, 24 states required 12‐month continuous Medicaid coverage for children before redetermining eligibility, while 26 states and the District of Columbia had no such requirement; the states without the 12‐month continuous coverage requirement could conduct periodic eligibility checks at least once or more frequently in addition to the mandatory check for annual renewal [12, 13]. Redetermining eligibility frequently could disrupt coverage due to temporary income or other eligibility changes [14]. Previous research suggests that low‐income children in states with 12‐month continuous Medicaid coverage were more likely to have Medicaid coverage, were less likely to experience insurance gaps, and had better access to care compared with children in states without this policy [4, 15].

Between March 2020 and March 2023, the Families First Coronavirus Response Act (FFCRA) required states to offer continuous Medicaid coverage to beneficiaries in exchange for increased federal Medicaid funding. This policy prohibited states from redetermining Medicaid eligibility during this period, effectively offering continuous Medicaid coverage to enrolled beneficiaries regardless of changes in their income or categorical eligibility. Emerging evidence suggests this provision increased Medicaid enrollment for non‐elderly adults [16] and postpartum women [17, 18], reduced coverage gaps for low‐income adults [11, 19, 20, 21], and improved access among adult Medicaid beneficiaries [19].

There is less evidence on how the FFCRA Medicaid continuity provision affected children. Some studies reported more children enrolled in Medicaid [16, 22, 23] and a decline in coverage interruptions [21] during 2020–2022. However, there is no direct evidence on how much of this change is among previously uninsured children versus those switching from private coverage, or whether this increase in Medicaid enrollment improved children's health care access and utilization and health status. Understanding these effects specifically in the states that had no prior coverage continuity requirements is important as they are most likely to have been affected by the FFCRA Medicaid continuity provision. Moreover, the Consolidated Appropriations Act of 2023 required all states to provide 12‐month continuous coverage for children in Medicaid and CHIP effective January 2024 [24]. Therefore, investigating the FFCRA effects on those states that did not previously have this 12‐month requirement provides an early assessment of the anticipated effects of this recent requirement.

This study examines the effects of the FFCRA Medicaid continuity provision on children's health insurance coverage, access to care, use of specific types of healthcare services, and health status in the states that had no prior continuity provisions by comparing them to states that required 12‐month continuous Medicaid coverage before the FFCRA. The study implements a difference‐in‐differences event‐study design that compares those two groups of states over time and estimates year‐by‐year effects following the FFCRA.

## 2 Methods

### 2.1 Data and Sample

Data come from the National Survey of Children's Health (NSCH) from 2016 through 2023. The NSCH is a nationally representative, cross‐sectional survey that selects each year one index child from each sampled household across all 50 states and the District of Columbia. The pooled data is, therefore, a repeated cross‐sectional sample. The NSCH samples households with children from the Census Bureau's Master Address File supplemented with administrative records to identify households with children [25]. Sampled households receive a mailed invitation including information about the NSCH and are asked to complete a screening questionnaire about the presence of children (ages 0–17) in the household, basic demographic information, and whether any children have special health care needs. Following screening, one child (from up to the first four reported children) is randomly selected from each eligible household for a detailed, age‐specific topical questionnaire about the child's physical and emotional health, access to and utilization of health care services, and sociodemographic characteristics of the child and household. Children with special health care needs and those aged 0–5 years are oversampled. A parent or caregiver most knowledgeable about the selected child's health completes the topical questionnaire; most surveys are completed by parents (nearly 92% in 2016–2023). The screening and topical questionnaires are self‐administered and can be completed either online or on paper and returned by mail. Survey staff can assist respondents via email and a toll‐free telephone number. Up to three follow‐up reminders are sent to increase response rates. Nearly 75% of sampled households eligible for the topical questionnaire complete the survey.

The analytical sample includes children aged 1–17 years. We exclude infants (age 0 years in the dataset) because newborns are automatically eligible for Medicaid coverage for 1 year if their mothers are enrolled in Medicaid at the time of delivery and, therefore, would not be affected by the FFCRA for the first 12 months [26]. We further limit the main sample to children with family income up to 300% of the federal poverty level (FPL) because this threshold exceeds mean Medicaid income eligibility for children aged 1–17 years across states (193% for children aged 1–5 years and 186% for children aged 6–17 years in 2019). Because the FFCRA maintained Medicaid coverage even if income after enrollment exceeded eligibility, it is important to allow for income fluctuations that exceed the eligibility threshold. Moreover, since family income in the NSCH is reported for the past calendar year, this cutoff also allows for income declines that would make a child newly eligible in the current survey year.

We also consider two alternate samples as a sensitivity check and to further account for eligibility differences across states. In 2019, children's Medicaid income eligibility across states ranged between 155% and 324% FPL. Therefore, the proportion of children who would benefit from the FFCRA's continuous Medicaid coverage varies between states based on the eligibility threshold. The first alternate sample includes children in households with income up to the Medicaid eligibility threshold in the state. An issue with this sample, however, is that it excludes children who would have become eligible for Medicaid later due to a decline in family income, such as parental job loss, which occurred frequently earlier in the COVID‐19 pandemic. The second sample includes children up to that threshold plus 100% to allow for income fluctuations after enrollment and from the last calendar year.

### 2.2 Outcomes

We examine outcomes related to children's health insurance coverage, access to care, health services utilization, and health status. We include five binary (0/1) measures of children's health insurance at the survey time: public coverage (non‐military), any private coverage, employer‐sponsored coverage, privately purchased coverage, and any insurance coverage. We examine private coverage outcomes to check for any crowd‐out effects from private to public coverage. We also evaluate a binary measure of whether the child experienced any insurance gaps in the past 12 months. For access to care, we include three binary measures for whether the child has: a usual place for sick care; a usual place for preventive care; and one or more persons who serve as the child's personal doctor or nurse. Another access outcome is a binary indicator for whether the child had any unmet health care needs in the past 12 months.

We include six binary measures of using specific health services over the past 12 months, including any medical visits, any preventive visits, any mental health visits, any specialist visits, any emergency department visits, and any hospital admissions (measured beginning in 2018). Health status is measured by parent/caregiver rating of the child's overall health status on a five‐category scale (poor, fair, good, very good, or excellent) which we evaluate in two binary measures: excellent/very good versus good/fair/poor health, and poor/fair versus good/very good/excellent health.

### 2.3 Statistical Analysis

We employ a difference‐in‐differences event study design that compares outcome differences over time (year‐by‐year) before and after the FFCRA enactment between states that did not have 12‐month continuous Medicaid coverage in 2019 (treatment group) and states that already had this policy (control group), with 2019 as the reference year. The control states implemented their 12‐month continuous coverage policies much earlier than the FFCRA. Of the 24 control states, 16 states enacted their own continuous Medicaid coverage before 2008 [15], 7 states between 2008 and 2010 [15], and 1 state in 2014 [27]. Therefore, the effects of these state requirements of 12‐month continuous coverage would likely have stabilized before 2016, the first year of our study period, and are unlikely to contribute to differences in trends between the treatment and control states over the study period.

We estimate the following regression model to examine the effects of the FFCRA continuous Medicaid coverage:

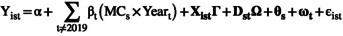


$Y_{\mathrm{ist}}$ is one of the study outcomes described above for child i in state s interviewed in year t. $MC_{s}$ is a binary indicator that equals 1 if the state did not have its own 12‐month continuous Medicaid coverage for children in 2016–2019 (i.e., the treatment group), and 0 if the state had this coverage (i.e., the control group). Table S1 lists the assignment of states into treatment and control groups. As noted above, all states that had their own 12‐month continuous coverage had this provision before 2016 so there is no change in that policy in the study period. $\mathrm{Yea}r_{t}$ is a set of binary indicators for each year from 2016 to 2023, except for 2019 as the reference year. $X_{\mathrm{ist}}$ includes child's age (0/1 indicators for each age in years), sex, race/ethnicity (non‐Hispanic Black, non‐Hispanic White, non‐Hispanic other races, and Hispanic), total number of children living in the household, highest educational level of parents/caregivers (less than high school, high school, some college, and college or above), household income as percentage of FPL in categories (below 100% FPL, 100–199% FPL, and 200–300% FPL), parental marital status, whether anyone in the household is employed, and the primary household language (English, Spanish, and other languages). $D_{\mathrm{st}}$ includes two state‐level time‐varying covariates—whether the state had expanded Medicaid under the Affordable Care Act (ACA) by year t and unemployment rate. The model also includes binary indicators for state of residence in $\theta_{s}$ to adjust for time‐invariant differences between states, and for year in $\omega_{t}$ to capture national trends shared across states. In the sensitivity analyses using the alternate samples selecting an income sample from each state specific to the state's eligibility, the model adds interactions between income categories and state fixed effects to account for varying income effects across states, as well as interactions between income and year fixed effects to capture different outcome trends by income over time.

In the above regression, coefficients $\beta_{2020},\beta_{2021},\beta_{2022}$ and $\beta_{2023}$ estimate the FFCRA effects in 2020, 2021, 2022, and 2023, respectively. Because the FFCRA started in March of 2020 and ended in March 2023 and because the NSCH is administered in the second half of the year, we would expect the FFCRA effects on coverage, access, and health services use to be more pronounced in 2021 and 2022 especially for outcomes measured over the past 12 months. Coefficients $\beta_{2016}$, $\beta_{2017}$, and $\beta_{2018}$ capture differences in pre‐FFCRA trends between the treatment and control groups (in 2016, 2017, and 2018, respectively, vs. 2019); significant differences in pre‐trends would raise concerns about the validity of the difference‐in‐differences design, that is, the parallel trends assumption (PTA). To evaluate the sensitivity of estimates to any violations of PTA, we follow the approach by Rambachan and Roth [28]. Specifically, for any statistically significant post‐FFCRA estimates under the difference‐in‐differences PTA, we construct 95% confidence bounds that allow for deviations from strictly parallel trends based on smoothness restrictions on how the slope of the differential trend between the treatment and control groups changes over time.

The regression is estimated using ordinary least squares weighted by NSCH sampling weights to obtain directly interpretable and nationally representative effect estimates. We report 95% confidence intervals (CIs) based on standard errors clustered at the state level. Estimates are considered statistically significant at the 5% level. All statistical analyses are conducted using StataNow SE/18.5.

## 3 Results

### 3.1 Sample Description

Depending on the outcome, the main analytical sample ranges from 122,901 to 126,117 children. Table 1 provides summary statistics for the treatment and control groups before and after FFCRA enactment. During pre‐FFCRA years (2016–2019), treatment states had lower rates of public coverage (50.8 vs. 55.7%), having a usual place for preventive care (87.9 vs. 89.4%), having a personal doctor or nurse (64.4 vs. 67.8%), and preventive check‐ups (74.9 vs. 76.0%), and higher rates of experiencing insurance gaps (14.1 vs. 8.8%) and unmet care needs (4.6 vs. 3.5%). Sociodemographic characteristics were relatively close between the treatment and control groups. Treatment states were less likely to have expanded Medicaid under the Affordable Care Act (ACA; 37.9% of the treatment sample vs. 80.0% of the control sample).

**TABLE 1:** Main Sample Descriptive Statistics.

	2016–2019		2020–2023	
	States with 12‐month continuous coverage requirement pre‐FFCRA (Control)	States without continuous coverage requirement pre‐FFCRA (Treatment)	States with 12‐month continuous coverage requirement pre‐FFCRA (Control)	States without continuous coverage requirement pre‐FFCRA (Treatment)
*OUTCOMES*				
Health insurance				
Public coverage	55.74%	50.75%	56.64%	50.48%
Any private coverage	43.98%	44.38%	42.99%	45.14%
Employer‐Sponsored coverage	39.26%	38.49%	37.41%	38.72%
Privately purchased coverage	5.97%	6.61%	5.63%	5.78%
Any insurance coverage	93.79%	89.54%	92.83%	88.97%
Insurance gap	8.82%	14.13%	8.80%	12.95%
Access to care				
Usual place for sick care	74.51%	73.93%	70.97%	71.49%
Usual place for preventive care	89.39%	87.91%	86.70%	86.44%
Have a personal doctor	67.79%	65.41%	65.35%	64.73%
Unmet care needs	3.45%	4.61%	4.59%	4.87%
Health services use				
Any medical visits	79.82%	78.67%	77.32%	77.72%
Any preventive check‐up	76.02%	74.95%	72.56%	73.31%
Any mental visits	8.06%	8.71%	9.92%	9.62%
Any specialist visits	11.94%	12.40%	10.43%	10.89%
Any ED visits	22.10%	22.61%	17.44%	18.59%
Any hospital stay [1]	4.13%	3.46%	2.99%	2.74%
Parent perceived child health status				
Excellent/very good health	87.29%	86.63%	86.66%	87.28%
Poor/fair health	1.75%	2.17%	2.11%	2.02%
*COVARIATES*				
Child's age				
1–5 Years Old	28.66%	28.96%	27.68%	27.45%
6–11 Years Old	36.31%	36.93%	34.86%	35.50%
12–17 Years Old	35.03%	34.11%	37.45%	37.05%
Child's sex				
Male	49.75%	52.08%	50.72%	51.70%
Female	50.25%	47.92%	49.28%	48.30%
Child's race/ethnicity				
Non‐Hispanic White	40.30%	42.21%	38.96%	41.42%
Non‐Hispanic Black	15.08%	16.99%	14.95%	15.90%
Non‐Hispanic Others	10.27%	8.83%	11.15%	9.53%
Hispanic	34.35%	31.97%	34.93%	33.16%
Highest education in household				
Less than High School	14.21%	13.19%	14.71%	12.48%
High School	26.97%	28.80%	28.09%	28.44%
Some College	29.35%	27.68%	25.80%	26.76%
College and above	29.48%	30.33%	31.40%	32.31%
Number of kids in household				
1	22.26%	22.17%	22.33%	22.15%
2	35.61%	34.54%	36.42%	35.76%
3	25.58%	26.09%	24.84%	25.96%
4+	16.55%	17.20%	16.41%	16.14%
Household income as percentage of FPL				
Below 100% FPL	31.50%	31.04%	29.96%	28.63%
100%–199% FPL	39.66%	38.81%	37.51%	37.77%
200%–300% FPL	28.83%	30.14%	32.52%	33.60%
Parental marital status				
Married	75.92%	75.14%	75.39%	75.66%
Not married	24.08%	24.86%	24.61%	24.34%
At least one employee in household				
Yes	8.45%	8.54%	5.42%	4.77%
No	91.55%	91.46%	94.58%	95.23%
Primary household language				
English	78.95%	79.41%	75.71%	79.28%
Spanish	15.64%	15.55%	18.55%	15.87%
Other	5.41%	5.03%	5.74%	4.85%
State‐level covariates				
State Unemployment Rate	4.47%	3.99%	5.62%	4.75%
State Medicaid Expansion Status	80.22%	37.34%	81.70%	47.50%
*N*	23,322	25,302	41,488	36,005

*Note:* The sample includes children aged 1–17 years in families with income below or at 300% federal poverty level. The descriptive statistics are weighted by the NSCH sampling weights [1]. Data on any hospital stays was first collected in 2018 and is not available in 2016 and 2017.

Abbreviations: FFCRA, Families First Coronavirus Response Act; FPL, federal poverty level.

### 3.2 Effects on Health Insurance Coverage

**FIGURE 1:**
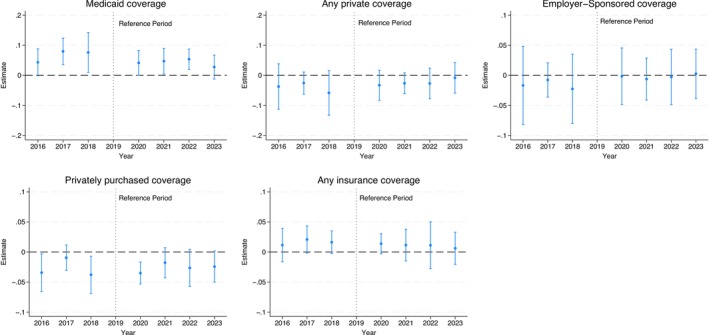
Difference‐in‐differences event study estimates of the FFCRA medicaid continuity effects on children's health insurance coverage. 95% confidence intervals are in brackets and derived from state‐clustered standard errors. The sample includes children aged 1–17 years in families with income up to 300% federal poverty level. The estimates are obtained from a difference‐in‐difference event‐study regression that estimates year‐by‐year differences in outcomes from 2016 to 2023 versus 2019 as the reference year between the treatment and control groups. All models adjust for age, sex, race/ethnicity, total number of children living in the household, highest educational level of parents/caregivers, marital status, any household employment, family income, state Medicaid expansion status, state unemployment rate, survey year, and state of residence. All models are weighed by the NSCH sampling weights to yield nationally representative estimates. FFCRA, Families First Coronavirus Response Act; NSCH, National Survey of Children's Health.

Figure 1 reports the difference‐in‐differences estimates of the FFCRA effects on health insurance coverage (see Table S2 for detailed estimates). In states without pre‐FFCRA 12‐month Medicaid continuity, public coverage increased in 2020, 2021, and 2022 by 4.1 (95% CI: 0.004–8.3), 4.7 (95% CI: 0.4–9.0), and 5.4 (95% CI: 2.0–8.7) percentage points, respectively, versus 2019, compared to states that had a pre‐FFCRA 12‐month Medicaid continuity requirement. The increase in 2023 is smaller and not statistically significant. There are no statistically significant effects on any private coverage, employer‐sponsored coverage, and any coverage, but there is a decline in privately purchased coverage in 2020 by 3.5 (95% CI: −5.3 to −1.7) percentage points. Similarly, there are no significant effects on experiencing gaps in the past 12 months.

Pre‐FFCRA trends show a decline in public coverage rates in treatment states from 2017 to 2018 to 2019 compared to control states (consistent with the unadjusted trends in Figure S1). Even though the 2017 and 2018 coefficients (relative to 2019) are of the same sign (positive) as the 2020–2022 coefficients (also relative to 2019), these pre‐ and post‐trend changes are in the opposite direction. Specifically, the positive coefficients for outcome differences between 2017 and 2018 and 2019 in treatment versus control states indicate that the treatment states had lower public coverage in 2019 than 2017–2018 relative to the control states. In contrast, the positive coefficients in 2020–2022 versus 2019 indicate that the treatment states had an increase in public coverage rates from 2019 to 2020 to 2022 compared to the control states. The reversal of the pre‐FFCRA decline in public coverage in the treatment states following the FFCRA is consistent with the intended effect of this policy. We discuss potential contributors to this pre‐trend difference between the treatment and control states below. Privately purchased coverage rates in treatment states were also higher in 2019 than in 2018 and 2016 in the treatment states; this pre‐FFCRA difference is also in the opposite direction to the decline from 2019 to 2020.

To assess the sensitivity of estimates to potential violations of the PTA, Figure S2 shows the confidence bounds for the FFCRA effects on coverage that allow for deviations from strictly parallel trends, following the approach proposed by Rambachan and Roth [28]. The effects indicating increased public coverage in 2021 and 2022 are robust to deviations of parallel trends under most examined scenarios. However, the 2020 estimates for public coverage and privately purchased coverage are not robust to small deviations and should, therefore, be interpreted with caution.

### 3.3 Effects on Access to Care

**FIGURE 2:**
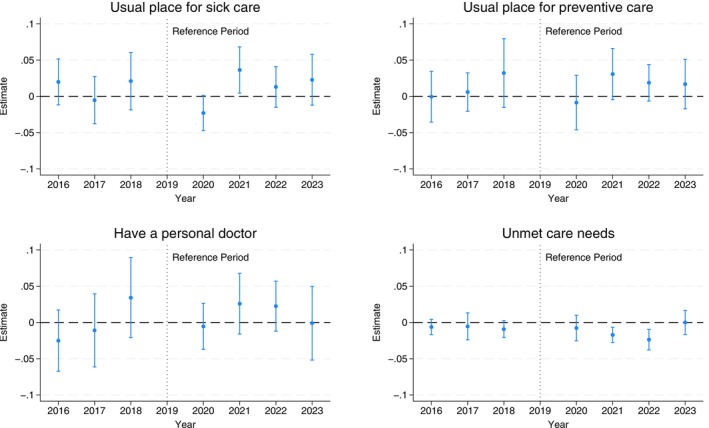
Difference‐in‐differences event study estimates of the FFCRA medicaid continuity on children's access to care. 95% confidence intervals are in brackets and derived from state‐clustered standard errors. The sample includes children aged 1–17 years in families with income up to 300% federal poverty level. The estimates are obtained from a difference‐in‐difference event‐study regression that estimates year‐by‐year differences in outcomes from 2016 to 2023 versus 2019 as the reference year between the treatment and control groups. All models adjust for age, sex, race/ethnicity, total number of children living in the household, highest educational level of parents/caregivers, marital status, any household employment, family income, state Medicaid expansion status, state unemployment rate, survey year, and state of residence. All models are weighed by the NSCH sampling weights to yield nationally representative estimates. FFCRA, Families First Coronavirus Response Act; NSCH, National Survey of Children's Health.

Figure 2 presents the difference‐in‐differences estimates for access to care; detailed estimates are in Table S3. Most estimates are small and not statistically significant. There is an increase in the likelihood of a usual place for sick care by 3.6 (95% CI: 0.5 to 6.8) percentage points in 2021 and a decrease in unmet care needs in 2021 and 2022 by 1.7 (95% CI: −2.8 to −0.7) and 2.4 (95% CI: −3.8 to −1.0) percentage points, respectively. There are no significant pre‐trends based on the event study estimates, consistent with the unadjusted trends (Figure S3). Figure S4 shows the confidence bounds for effects on access to care outcomes, assessing sensitivity to potential PTA violations. The effect estimate for a usual place for sick care in 2021 is not robust to any deviations from parallel trends and should, therefore, be viewed with caution. The 2021 and 2022 effect estimates for unmet care needs are robust to a linear difference in trends, and the latter estimate is robust to a small non‐linear deviation over time, although not to larger changes.

### 3.4 Effects on the Likelihood of Using Specific Types of Health Services

**FIGURE 3:**
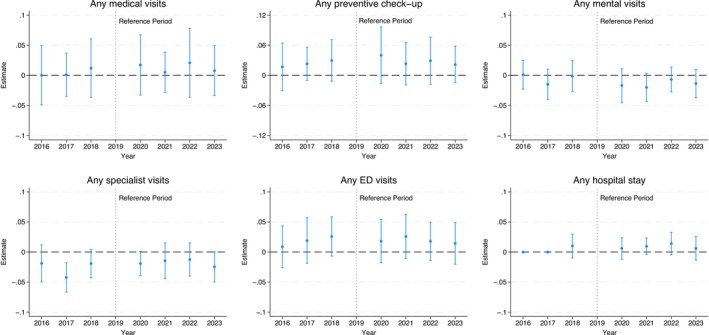
Difference‐in‐differences event study estimates of the FFCRA medicaid continuity effects on children's use of health services by type. 95% confidence intervals are in brackets and derived from state‐clustered standard errors. The sample includes children aged 1–17 years in families with income up to 300% federal poverty level. The estimates are obtained from a difference‐in‐difference event‐study regression that estimates year‐by‐year differences in outcomes from 2016 to 2023 versus 2019 as the reference year between the treatment and control groups. The 2016 and 2017 estimates for any hospital stay have been suppressed because no data are available for these years. All models adjust for age, sex, race/ethnicity, total number of children living in the household, highest educational level of parents/caregivers, marital status, any household employment, family income, state Medicaid expansion status, state unemployment rate, survey year, and state of residence. All models are weighted by the NSCH sampling weights to yield nationally representative estimates. FFCRA, Families First Coronavirus Response Act; NSCH, National Survey of Children's Health; ED, emergency department.

Figure 3 shows the difference‐in‐differences estimates for the likelihood of using specific types of health services (see Table S4 for detailed estimates). Most estimates are small, and none are statistically significant. Pre‐trend differences are generally small, and most are not statistically significant, consistent with the unadjusted trends (Figure S5).

### 3.5 Effects on Health Status

**FIGURE 4:**
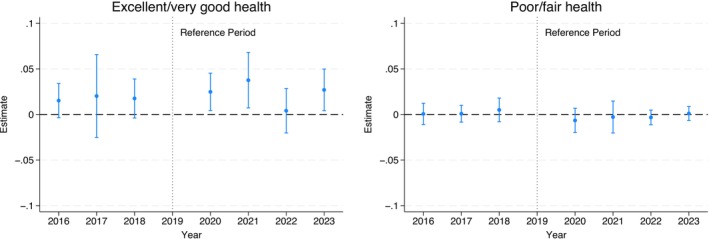
Difference‐in‐differences event study estimates of the FFCRA medicaid continuity effects on children's health status as rated by parents/caregivers. 95% confidence intervals are in brackets and derived from state‐clustered standard errors. The sample includes children aged 1–17 years in families with income up to 300% federal poverty level. The estimates are obtained from a difference‐in‐difference event‐study regression that estimates year‐by‐year differences in outcomes from 2016 to 2023 versus 2019 as the reference year between the treatment and control groups. All models adjust for age, sex, race/ethnicity, total number of children living in the household, highest educational level of parents/caregivers, marital status, any household employment, family income, state Medicaid expansion status, state unemployment rate, survey year, and state of residence. All models are weighed by the NSCH sampling weights to yield nationally representative estimates. FFCRA, Families First Coronavirus Response Act; NSCH, National Survey of Children's Health.

Figure 4 presents the difference‐in‐differences estimates for health status (see Table S5 for detailed estimates). There is a greater likelihood of excellent/very good health in 2020, 2021, and 2023 by 2.5 (95% CI: 0.4 to 4.5), 3.8 (95% CI: 0.7 to 6.8), and 2.7 (95% CI: 0.4 to 5.0) percentage points, respectively, in the treatment states. The estimate for 2022 is small and not statistically significant. Estimates for poor/fair health are not statistically significant. There are no significant pre‐trends, consistent with the unadjusted trends (Figure S6). Figure S7 shows confidence bounds considering potential PTA violations. Estimates for the increase in excellent/very good health in 2020, 2021, and 2023 are robust to a linear trend difference, and the estimate in 2021 is robust to some non‐linear differences.

### 3.6 Estimates From Alternate Samples

There are no effects on public coverage when limiting the sample to the state's Medicaid income eligibility level (Table S6). These effects are more pronounced when adding children up to 100% above the state's Medicaid eligibility level, but only the increase in 2022 is statistically significant. Similar to the main sample, there is a decline in unmet care needs in 2021 and 2022 (Table S7), little evidence of effects on the likelihood of using specific types of services (Table S8) and an increase in excellent/very good health in 2020, 2021, and 2023 (Table S9).

## 4 Discussion

This study examines the effects of the FFCRA Medicaid coverage continuity on children's health insurance, access to care, likelihood of using specific types of health services, and health status in states that did not have a Medicaid continuity provision before the FFCRA (treatment group) by comparing them to states that previously had a 12‐month continuous Medicaid coverage provision (control group). The FFCRA increased public coverage in the treatment states. However, some of these increases appear to be shifts from private coverage to public coverage, as there is no apparent effect on any coverage. Finding no change in insurance gaps further suggests switching from private to public coverage. There is some evidence of an increase in having a usual source of sick care in 2021 and a decline in unmet care needs in 2021 and 2022 in the treatment states. However, there are no apparent effects on the likelihood of preventive check‐ups, mental health visits, emergency department visits, or hospitalizations. Lastly, there is an improvement in children's health status rating as excellent or very good. Most of these findings are robust to at least a linear pre‐trend difference between treatment and control states, except for the increase in public coverage and decline in private coverage in 2020, and having a usual place for sick care in 2021. The inference on public coverage increases in 2021–2022 and decline in unmet care needs in 2022 is also robust to small non‐linear differences in trends.

The increase in public coverage aligns with other studies reporting more children covered under Medicaid in states with pre‐FFCRA 12‐month continuity or during the FFCRA [4, 23]. Using monthly Medicaid enrollment data, Vasan et al. reported a nearly 2 percentage‐point increase in total Medicaid enrollment in March 2021 among states without pre‐FFCRA continuous coverage provision [23]. Our estimates are likely larger because our sample focuses on low‐income children. We observe some pre‐trend differences between the treatment and control states in public insurance coverage—treatment states had a decline in public coverage from 2017 to 2018 to 2019 relative to control states—although inference is generally robust to this difference as noted above. A potential explanation for this pre‐trend difference is federal and state policy changes beginning in 2017 that increased administrative burdens for Medicaid enrollment and renewal, including more stringent and frequent eligibility verification and automatic disenrollment following renewal requests mailed by state Medicaid agencies that receive no response [12, 29, 30]. A recent study reported that these administrative changes reduced children's Medicaid/CHIP enrollment nationally by up to 1.7 percentage points [30]. That study did not consider potential heterogeneity by whether the states had their own 12‐month coverage continuity. However, it is possible that these added administrative burdens had a more pronounced negative effect on Medicaid enrollment in the states without 12‐month continuous coverage (the treatment group in our study) than in the control states, which might explain the pre‐trend difference in public coverage we observed. The timing of these administrative changes aligns with the observed pre‐trend difference in our event study.

Finding no effect on overall insurance rates is similar to another study through 2020 and suggests switching from private to public coverage [21]. This switching likely reflects the pandemic disruptions to labor markets and subsequent parental loss of jobs and private coverage. Indeed, in the alternate sample that only includes children with family income in the past calendar year up to the income eligibility level, there are no apparent effects on public coverage. In contrast, we observe an increase in public coverage when the income range exceeds the income eligibility level, suggesting that some children who were previously income ineligible may have become income eligible over time due to subsequent income decline. The decline in unmet care needs in 2021 and 2022 may reflect improvement in care continuity with longer enrollment in Medicaid. Even for children who switched from private to public coverage, access may improve with no or much lower out‐of‐pocket cost sharing (premiums or copayments) than private coverage. The effect on unmet care needs dissipates in 2023, similar to the effect on coverage, consistent with the unwinding of continued Medicaid coverage in 2023.

Notably, there is improvement in reporting excellent/very good health in all years except 2022, which might reflect the accumulating effects of improved access over time. These improvements in children's general health status are also comparable to previous studies that demonstrated similar increases in health status following the Medicaid/CHIP expansions or among children with better insurance continuity [2, 9, 10, 31, 32, 33]. This finding is also consistent with studies that found improvement in the general health status of low‐income adults following the ACA Medicaid income‐eligibility expansions [34, 35, 36].

Although we find some increase in public coverage and decline in unmet care needs, estimates suggest overall no changes in the use of certain types of health services. One possible reason is that NSCH questions capture any use of services within the past 12 months, rather than the intensity or frequency of utilization. Coverage continuity may influence care delays and the frequency and intensity of services utilization rather than any use. If coverage continuity influences more the frequency and intensity of services rather than any use (especially for children with at least some prior coverage and access), such effects would not be captured by the available measures of any utilization, although they would be captured in changes in unmet care needs. In addition, as mentioned above, there is some potential switching from private to public coverage. For those children, Medicaid coverage reduces out‐of‐pocket cost‐sharing, which may also improve access to care by reducing unmet care needs without necessarily increasing outcomes based on any utilization of health services. With less out‐of‐pocket cost, there could be less delay in getting care, more visits, or more intensive treatment. The decline in out‐of‐pocket cost may, therefore, reduce unmet care needs without meaningfully changing measures of any health care use that do not capture treatment delay, frequency, or intensity. Finally, it is likely that parent/caregiver reported health care use measures in NSCH were not sensitive enough to capture the shift from in‐person visits to virtual visits [37, 38] that occurred during the pandemic. Although the NSCH questionnaire added a note reminding respondents to include telehealth visits, this note was added from 2021 onwards and only for the question any medical visits (one of our studied outcomes).

Our findings suggest that extending children's Medicaid coverage continuity for at least 12 months brings some benefits to accessing care and potentially health status. Because the control group already had 12‐month continuous Medicaid coverage and the FFCRA extended coverage longer than 12 months, comparing the treatment and control groups captures two effects. The first is the effect of providing 12‐month continuity versus no continuity. The second effect is any difference in the effects of extending coverage longer than 12 months between the treatment and control groups. It is not possible to separate those two effects. However, this potential second effect would likely increase over time with a longer duration of continuous coverage. If continuity longer than 12 months affects children more in the treatment states, the difference‐in‐differences effect estimates would increase with later years. We generally observe effects beginning in 2020 or 2021 without clear evidence of intensifying effects subsequently. It is possible that longer continuity improved outcomes similarly in both groups (i.e., null second effect), in which case the difference‐in‐differences estimates would primarily reflect the effect of providing 12‐month coverage continuity compared to no coverage continuity. The unadjusted outcome trends show an increase in preventive checkups, specialist visits, and ED visits in both the treatment and control groups (Figure S4), which might suggest improvement in these outcomes in the states with pre‐FFCRA 12‐month continuity and may be another factor that explains the lack of effects on health services utilization. This descriptive interpretation, however, should be cautiously considered.

Taken as a whole, the findings suggest that providing Medicaid coverage continuity for children for at least 12 months may improve some access measures and potentially health status. As such, the new federal requirement that all states provide 12‐month continuous coverage for children in Medicaid and CHIP beginning January 2024 may improve access in the states that did not have this requirement [23, 24]. Understanding the effects of providing longer coverage than 12 months before redetermining remains an open question that deserves more research especially as more states explore such extensions [39].

### 4.1 Limitations

Study strengths include nationally representative data, multiple measures on health insurance coverage, access to care, specific types of health services use and health status, and a difference‐in‐difference event‐study design that accounts for contemporaneous confounding events. One limitation is that we cannot separate children covered by Medicaid from those covered by a separate CHIP who were not subject to the continuous Medicaid coverage requirement, which could have attenuated effect estimates [40]. Also, the unmet care and health service outcomes are based on the past 12 months and can span two calendar years. The NSCH data does not include the survey month. For children in 2020 and 2023, we are not able to account for the length of exposure to the FFCRA in these outcomes [41]. Moreover, we cannot account for seasonality effects. However, these concerns are partially mitigated by the NSCH data collection schedule, which occurs in the second half of each year (up to January of the following year). Moreover, this limitation affects all years and both treatment and control states, and therefore, is unlikely to meaningfully affect the difference‐in‐differences estimates. Also, as noted above, another limitation is that the health services measures capture any utilization rather than intensity or frequency. Future research with other data can examine more granular utilization measures. Lastly, it is worth noting that states likely differed in how they implemented the renewal process during the unwinding of the FFCRA in 2023 including the duration to complete all redeterminations of eligibility, employing automatic renewals, and outreach to and communication with beneficiaries [42]. Understanding potential heterogeneity in effects of the continuous coverage unwinding based on such state policies and strategies is important for future research.

## Disclosure

The authors have nothing to report.

## Conflicts of Interest

The authors declare no conflicts of interest.

## Supporting information


**Data S1:** Supporting Information.

## Data Availability

The data that support the findings of this study are available in United States Census Bureau at https://www.census.gov/programs‐surveys/nsch.html. These data were derived from the following resources available in the public domain: NSCH Data, https://www.census.gov/programs‐surveys/nsch/data/datasets.html.

## References

[1] A. Cassedy , G. Fairbrother , and P. W. Newacheck , “The Impact of Insurance Instability on Children's Access, Utilization, and Satisfaction With Health Care,” Ambulatory Pediatrics 8, no. 5 (2008): 321–328. 18922506 10.1016/j.ambp.2008.04.007

[2] A. Osorio and J. Alker , “Kids with Gaps in Coverage Have Less Access to Care. Center for Children and Families, McCourt School of Public Policy, Georgetown University,” accessed May 10, 2025, https://ccf.georgetown.edu/2021/10/15/kids‐with‐gaps‐in‐coverage‐have‐less‐access‐to‐care.

[3] L. J. Leininger , “Partial‐Year Insurance Coverage and the Health Care Utilization of Children,” Medical Care Research and Review 66, no. 1 (2009): 49–67, 10.1177/1077558708324341. 18981264

[4] E. Brantley and L. Ku , “Continuous Eligibility for Medicaid Associated With Improved Child Health Outcomes,” Medical Care Research and Review 79, no. 3 (2022): 404–413. 34525877 10.1177/10775587211021172

[5] L. M. Olson , S. F. Tang , and P. W. Newacheck , “Children in the United States With Discontinuous Health Insurance Coverage,” New England Journal of Medicine 353, no. 4 (2005): 382–391, 10.1056/NEJMsa043878. 16049210

[6] L. A. Blewett , G. Davidson , M. D. Bramlett , H. Rodin , and M. L. Messonnier , “The Impact of Gaps in Health Insurance Coverage on Immunization Status for Young Children,” Health Services Research 43, no. 5 Pt 1 (2008): 1619–1636, 10.1111/j.1475-6773.2008.00864.x. 18522671 PMC2653891

[7] N. J. Allred , K. G. Wooten , and Y. Kong , “The Association of Health Insurance and Continuous Primary Care in the Medical Home on Vaccination Coverage for 19‐ to 35‐Month‐Old Children,” Pediatrics 119, no. Supplement_1 (2007): S4–S11, 10.1542/peds.2006-2089C. 17272584

[8] J. E. DeVoe , A. Graham , L. Krois , J. Smith , and G. L. Fairbrother , ““Mind the Gap” in Children's Health Insurance Coverage: Does the Length of a Child's Coverage Gap Matter?,” Ambulatory Pediatrics 8, no. 2 (2008): 129–134. 18355742 10.1016/j.ambp.2007.10.003 PMC4918900

[9] C. Gushue , R. Miller , S. Sheikh , et al., “Gaps in Health Insurance Coverage and Emergency Department Use Among Children With Asthma,” Journal of Asthma 56, no. 10 (2019): 1070–1078, 10.1080/02770903.2018.1523929. 30365346

[10] A. B. Bindman , A. Chattopadhyay , and G. M. Auerback , “Medicaid Re‐Enrollment Policies and Children's Risk of Hospitalizations for Ambulatory Care Sensitive Conditions,” Medical Care 46, no. 10 (2008): 1049–1054, 10.1097/MLR.0b013e318185ce24. 18815526

[11] S. Sugar , C. Peters , N. D. Lew , and B. D. Sommers , “Medicaid Churning and Continuity of Care: Evidence and Policy Considerations Before and After the COVID‐19 Pandemic. Office of the Assistant Secretary for Planning and Evaluation, U.S. Department of Health and Human Services,” accessed May 11, 2025, https://aspe.hhs.gov/sites/default/files/private/pdf/265366/medicaid‐churning‐ib.pdf.

[12] S. Artiga and O. Pham , “Recent Medicaid/CHIP Enrollment Declines and Barriers to Maintaining Coverage. KFF,” accessed May 11, 2025, https://www.kff.org/medicaid/issue‐brief/recent‐medicaid‐chip‐enrollment‐declines‐and‐barriers‐to‐maintaining‐coverage/.

[13] T. Brooks , L. Roygardner , and S. Artiga , “Medicaid and CHIP Eligibility, Enrollment, and Cost Sharing Policies as of January 2019: Findings from a 50‐State Survey. KFF,” accessed May 11, 2025, https://www.kff.org/medicaid/report/medicaid‐and‐chip‐eligibility‐enrollment‐and‐cost‐sharing‐policies‐as‐of‐january‐2019‐findings‐from‐a‐50‐state‐survey/.

[14] K. Swartz , P. F. Short , D. R. Graefe , and N. Uberoi , “Reducing Medicaid Churning: Extending Eligibility for Twelve Months or to End of Calendar Year Is Most Effective,” Health Aff (Millwood) 34, no. 7 (2015): 1180–1187. 26153313 10.1377/hlthaff.2014.1204 PMC4664196

[15] L. Ku , E. Steinmetz , and B. K. Bruen , “Continuous‐Eligibility Policies Stabilize Medicaid Coverage for Children and Could Be Extended to Adults With Similar Results,” Health Aff (Millwood) 32, no. 9 (2013): 1576–1582. 24019362 10.1377/hlthaff.2013.0362

[16] R. Sun , B. Staiger , A. Chan , L. C. Baker , and T. Hernandez‐Boussard , “Changes in Medicaid Enrollment During the COVID‐19 Pandemic Across 6 States,” Medicine 101, no. 52 (2022): e32487. 36596028 10.1097/MD.0000000000032487 PMC9803338

[17] U. Ejughemre , W. Lyu , and G. L. Wehby , “Effects of the Continuous Medicaid Coverage Provision of the Family First Coronavirus Response Act on Postpartum Medicaid Coverage, Depression Symptoms, and Birth Control Use,” Health Services Research 60 (2024): e14395, 10.1111/1475-6773.14395. 39402851 PMC12120518

[18] J. R. Daw , C. L. MacCallum‐Bridges , K. B. Kozhimannil , and L. K. Admon , “Continuous Medicaid Eligibility During the COVID‐19 Pandemic and Postpartum Coverage, Health Care, and Outcomes,” JAMA Health Forum 5, no. 3 (2024): e240004, 10.1001/jamahealthforum.2024.0004. 38457131 PMC10924249

[19] W. Lyu and G. L. Wehby , “Effects of the Families First Coronavirus Response Act on Coverage Continuity and Access for Medicaid Beneficiaries,” INQUIRY: The Journal of Health Care Organization, Provision, and Financing 61 (2024): 00469580241282052, 10.1177/00469580241282052. PMC11425735 39315678

[20] X. Wang , Y. M. Pengetnze , E. Eckert , G. Keever , and V. Chowdhry , “Extending Postpartum Medicaid Beyond 60 Days Improves Care Access and Uncovers Unmet Needs in a Texas Medicaid Health Maintenance Organization,” Frontiers in Public Health 10 (2022): 841832. 35592081 10.3389/fpubh.2022.841832 PMC9110670

[21] D. B. Nelson , A. L. Goldman , F. Zhang , and H. Yu , “Continuous Medicaid Coverage During the COVID‐19 Public Health Emergency Reduced Churning, but Did Not Eliminate It,” Health Affairs Scholar 1, no. 5 (2023): qxad055, 10.1093/haschl/qxad055. 38223316 PMC10786332

[22] A. Conmy , C. Peters , N. De Lew , and B. Sommers , “Children's health coverage trends: gains in 2020–2022 reverse previous coverage losses. Office of the Assistant Secretary for Planning and Evaluation, U.S. Department of Health and Human Services,” accessed May 11, 2025, https://aspe.hhs.gov/reports/childrens‐health‐coverage‐trends.

[23] A. Vasan , C. C. Kenyon , A. G. Fiks , and A. S. Venkataramani , “Continuous Eligibility and Coverage Policies Expanded Children's Medicaid Enrollment,” Health Aff (Millwood) 42, no. 6 (2023): 753–758, 10.1377/hlthaff.2022.01465. 37276479 PMC11299770

[24] E. Park , A. Dwyer , T. Brooks , M. Clark , and J. Alker , “Consolidated Appropriations Act, 2023: Medicaid and CHIP provisions explained. Center for Children and Families, McCourt School of Public Policy, Georgetown University,” accessed May 11, 2025, https://ccf.georgetown.edu/2023/01/05/consolidated‐appropriations‐act‐2023‐medicaid‐and‐chip‐provisions‐explained/.

[25] Census Bureau , “National Survey of Children's Health Methodology Report. U.S. Census Bureau,” 2021, accessed May 11, 2025, https://www2.census.gov/programs‐surveys/nsch/technical‐documentation/methodology/2021‐NSCH‐Methodology‐Report.pdf.

[26] Centers for Medicare Medicaid Services , “Pregnancy and Newborn Coverage in the Health Insurance Marketplace. Centers for Medicare Medicaid Services,” accessed May 11, 2025, https://www.cms.gov/marketplace/technical‐assistance‐resources/pregnancy‐newborn‐coverage.pdf.

[27] Colorado Department of Health Care Policy & Financing , “Continuous Eligibility Frequently Asked Questions. Colorado Department of Health Care Policy & Financing,” accessed May 13, 2025, https://hcpf.colorado.gov/sites/hcpf/files/Continuous%20Eligibility%20frequently%20asked%20questions_accessible.pdf.

[28] A. Rambachan and J. Roth , “A More Credible Approach to Parallel Trends,” Review of Economic Studies 90, no. 5 (2023): 2555–2591, 10.1093/restud/rdad018.

[29] T. Brooks , E. Park , and L. Roygardner , “Medicaid and CHIP enrollment decline suggests the child uninsured rate may rise again. Center for Children and Families, McCourt School of Public Policy, Georgetown University,” accessed May 11, 2025, https://ccf.georgetown.edu/2019/05/28/medicaid‐and‐chip‐enrollment‐decline/.

[30] I. Arbogast , A. Chorniy , and J. Currie , “Administrative Burdens and Child Medicaid and CHIP Enrollments,” American Journal of Health Economics 10, no. 2 (2024): 237–271. 40458769 10.1086/728170 PMC12129408

[31] M. Harrington , K. Smith , C. Trenholm , et al., “CHIPRA Mandated Evaluation of the Children's Health Insurance Program: Final Findings. Mathematica Policy Research,” accessed May 11, 2025, https://aspe.hhs.gov/sites/default/files/migrated_legacy_files/44396/rpt_CHIPevaluation.pdf.

[32] G. Kenney , “The Impacts of the State Children's Health Insurance Program on Children Who Enroll: Findings From Ten States,” Health Services Research 42, no. 4 (2007): 1520–1543, 10.1111/j.1475-6773.2007.00707.x. 17610436 PMC1955761

[33] E. M. Howell and C. Trenholm , “The Effect of New Insurance Coverage on the Health Status of Low‐Income Children in Santa Clara County,” Health Services Research 42, no. 2 (2007): 867–889, 10.1111/j.1475-6773.2006.00625.x. 17362222 PMC1890688

[34] J. Semprini , W. Lyu , D. M. Shane , and G. L. Wehby , “The Effects of ACA Medicaid Expansions on Health After 5 Years,” Medical Care Research and Review 79, no. 1 (2022): 28–35. 33218289 10.1177/1077558720972592 PMC8218574

[35] C. E. Margerison , C. L. MacCallum , J. Chen , Y. Zamani‐Hank , and R. Kaestner , “Impacts of Medicaid Expansion on Health Among Women of Reproductive Age,” American Journal of Preventive Medicine 58, no. 1 (2020): 1–11, 10.1016/j.amepre.2019.08.019. 31761513 PMC6925642

[36] B. D. Sommers , B. Maylone , R. J. Blendon , E. J. Orav , and A. M. Epstein , “Three‐Year Impacts of the Affordable Care Act: Improved Medical Care and Health Among Low‐Income Adults,” Health Aff (Millwood) 36, no. 6 (2017): 1119–1128, 10.1377/hlthaff.2017.0293. 28515140

[37] K. Schweiberger , A. Hoberman , J. Iagnemma , et al., “Practice‐Level Variation in Telemedicine Use in a Pediatric Primary Care Network During the COVID‐19 Pandemic: Retrospective Analysis and Survey Study,” Journal of Medical Internet Research 22, no. 12 (2020): e24345, 10.2196/24345. 33290244 PMC7752181

[38] K. N. Ray , S. R. Wittman , J. G. Yabes , L. M. Sabik , A. Hoberman , and A. Mehrotra , “Telemedicine Visits to Children During the Pandemic: Practice‐Based Telemedicine versus Telemedicine‐Only Providers,” Academic Pediatrics 23, no. 2 (2023): 265–270, 10.1016/j.acap.2022.05.010. 35589062 PMC9666718

[39] A. Diana , J. Tolbert , and A. Mudumala , “Medicaid and CHIP Eligibility Expansions and Coverage Changes for Children Since the Start of the Pandemic. KFF,” Updated 2025/01/26, accessed May 13, 2025, https://www.kff.org/medicaid/issue‐brief/medicaid‐and‐chip‐eligibility‐expansions‐and‐coverage‐changes‐for‐children‐since‐the‐start‐of‐the‐pandemic/.

[40] Centers for Medicare & Medicaid Services , “COVID‐19 Frequently Asked Questions (FAQs) for State Medicaid and Children's Health Insurance Program (CHIP) Agencies. Centers for Medicare & Medicaid Services,” accessed May 11th, 2025, https://www.medicaid.gov/state‐resource‐center/downloads/covid‐19‐faqs.pdf.

[41] A. S. Bernstein , S. Sun , K. R. Weinberger , K. R. Spangler , P. E. Sheffield , and G. A. Wellenius , “Warm Season and Emergency Department Visits to U.S. Children's Hospitals,” Environmental Health Perspectives 130, no. 1 (2022): 17001, 10.1289/ehp8083. 35044241 PMC8767980

[42] T. Brooks , A. Gardner , P. Yee , et al., “Medicaid and CHIP Eligibility, Enrollment, and Renewal Policies as States Prepare for the Unwinding of the Pandemic‐Era Continuous Enrollment Provision. KFF,” https://www.kff.org/medicaid/report/medicaid‐and‐chip‐eligibility‐enrollment‐and‐renewal‐policies‐as‐states‐prepare‐for‐the‐unwinding‐of‐the‐pandemic‐era‐continuous‐enrollment‐provision/.

