# Supplementary material for: Effects of Continuous Medicaid Coverage in 2020–2023 on Children's Health Insurance Coverage, Access to Care, Health Services Use by Type, and Health Status
Source: Health Serv Res. 2025 Aug 31;61(1):e70034. doi: 10.1111/1475-6773.70034 (PMC12857483; doi:10.1111/1475-6773.70034)
Supplement: Supplementary file 1 — Data S1: Supporting Information. [file HESR-61-e70034-s001.docx]

**Online Appendix**

[Appendix Table 1. State Assignment and Children’s Medicaid Income Eligibility 1](#_Toc204073637)

[Appendix Figure 1. Descriptive Unadjusted Trends of Children’s Health Insurance Coverage Outcomes between States with and without 12-Month Continuous Medicaid Coverage from 2016 to 2023 2](#_Toc204073638)

[Appendix Table 2. Difference-in-Differences Event Study Estimates of the FFCRA Medicaid Continuity Effects on Children’s Insurance coverage 3](#_Toc204073639)

[Appendix Figure 2. Confidence Bounds for the Estimated FFCRA Effects on Coverage Outcomes Accounting for Pre-trend Differences between Treatment and Control States 7](#_Toc204073640)

[Appendix Figure 3. Descriptive Unadjusted Trends of Children’s Access to Care Outcomes between States with and without 12-Month Continuous Medicaid Coverage from 2016 to 2023 8](#_Toc204073641)

[Appendix Table 3. Difference-in-Differences Event Study Estimates of the FFCRA Medicaid Continuity on Children’s Access to Care 9](#_Toc204073642)

[Appendix Figure 4. Confidence Bounds for the Estimated FFCRA Effects on Access to Care Outcomes Accounting for Pre-trend Differences between Treatment and Control States 13](#_Toc204073643)

[Appendix Figure 5. Descriptive Trends of Children’s Health Services Utilization Outcomes between States with and without 12-Month Continuous Medicaid Coverage from 2016 to 2023 14](#_Toc204073644)

[Appendix Table 4. Difference-in-Differences Event Study Estimates of the FFCRA Medicaid Continuity Effects on Children’s Health Services Utilization 15](#_Toc204073645)

[Appendix Figure 6. Descriptive Unadjusted Trends of Children’s Health Status as Rated by Parents/Caregivers between States with and without 12-Month Continuous Medicaid Coverage from 2016 to 2023 19](#_Toc204073646)

[Appendix Table 5. Difference-in-Differences Event Study Estimates of the FFCRA Medicaid Continuity Effects on Children’s Health Status as Rated by Parents/Caregivers 20](#_Toc204073647)

[Appendix Figure 7. Confidence Bounds for the Estimated FFCRA Effects on Excellent/Very Good Health Outcome Accounting for Pre-trend Differences between Treatment and Control States 24](#_Toc204073648)

[Appendix Table 6. Difference-in-Differences Event Study Estimates of the Federal Medicaid Continuous Coverage Requirement Effects on Children’s Health Insurance Coverage using Alternate Samples 25](#_Toc204073649)

[Appendix Table 7. Difference-in-Differences Event Study Estimates of the Federal Medicaid Continuous Coverage Requirement Effects on Children’s Access to Care using Alternate Samples 27](#_Toc204073650)

[Appendix Table 8. Difference-in-Differences Event Study Estimates of the Federal Medicaid Continuous Coverage Requirement Effects on Children’s Health Services Use using Alternate Samples 29](#_Toc204073651)

[Appendix Table 9. Difference-in-Differences Event Study Estimates of the Federal Medicaid Continuous Coverage Requirement Effects on Children’s Health Status as Rated by Parents/Caregivers using Alternate Samples 31](#_Toc204073652)

| Appendix Table 1. State Assignment and Children’s Medicaid Income Eligibility | | | | | | |
| --- | --- | --- | --- | --- | --- | --- |
| Control Group  (States with 12-month continuous Medicaid Coverage  before the FFCRA in 2019) | | |  | Treatment Group  (States without 12-month continuous Medicaid Coverage  before the FFCRA in 2019) | | |
| State | Medicaid Eligibility for Age 1-5 Years  (% of FPL) | Medicaid Eligibility  for Age 6-7 Years  (% of FPL) |  | State | Medicaid Eligibility for Age 1-5 Years  (% of FPL) | Medicaid Eligibility  for Age 6-7 Years  (% of FPL) |
| Alabama | 146 | 146 |  | Arizona | 146 | 138 |
| Alaska | 208 | 208 |  | Arkansas | 147 | 147 |
| California | 266 | 266 |  | Connecticut | 201 | 201 |
| Colorado | 147 | 147 |  | Delaware | 147 | 138 |
| Idaho | 147 | 138 |  | District of Columbia | 324 | 324 |
| Illinois | 147 | 147 |  | Florida | 145 | 138 |
| Iowa | 172 | 172 |  | Georgia | 154 | 138 |
| Kansas | 154 | 138 |  | Hawaii | 313 | 313 |
| Louisiana | 217 | 217 |  | Indiana | 165 | 165 |
| Maine | 162 | 162 |  | Kentucky | 164 | 164 |
| Michigan | 217 | 217 |  | Maryland | 322 | 322 |
| Mississippi | 148 | 138 |  | Massachusetts | 155 | 155 |
| Montana | 148 | 148 |  | Minnesota | 280 | 280 |
| New Jersey | 147 | 147 |  | Missouri | 155 | 155 |
| New Mexico | 305 | 245 |  | Nebraska | 218 | 218 |
| New York | 154 | 154 |  | Nevada | 165 | 138 |
| North Carolina | 215 | 138 |  | New Hampshire | 323 | 323 |
| North Dakota | 152 | 138 |  | Oklahoma | 210 | 210 |
| Ohio | 211 | 211 |  | Pennsylvania | 162 | 138 |
| Oregon | 138 | 138 |  | Rhode Island | 266 | 266 |
| South Carolina | 213 | 213 |  | South Dakota | 187 | 187 |
| Washington | 215 | 215 |  | Tennessee | 216 | 216 |
| West Virginia | 146 | 138 |  | Texas | 149 | 138 |
| Wyoming | 159 | 138 |  | Utah | 138 | 138 |
|  |  |  |  | Vermont | 317 | 317 |
|  |  |  |  | Virginia | 148 | 148 |
|  |  |  |  | Wisconsin | 191 | 156 |
| Notes: The information on the status of states’ 12-month continuous Medicaid coverage and Medicaid income eligibility for children is extracted from the Kaiser Family Foundation’s Annual Updates on Eligibility Rules, Enrollment and Renewal Procedures, and Cost-Sharing Practices in Medicaid and CHIP (available at: <https://www.kff.org/medicaid/report/annual-updates-on-eligibility-rules-enrollment-and-renewal-procedures-and-cost-sharing-practices-in-medicaid-and-chip/>). | | | | | | |

| Appendix Figure 1. Descriptive Unadjusted Trends of Children’s Health Insurance Coverage Outcomes between States with and without 12-Month Continuous Medicaid Coverage from 2016 to 2023 |
| --- |
|  |
| Notes: The sample includes children aged 1-17 years in families with income up to 300% federal poverty level. The statistics are all weighted by the NSCH sampling weights.  Abbreviation: FFCRA, Families First Coronavirus Response Act; NSCH, National Survey of Children’s Health |

| Appendix Table 2. Difference-in-Differences Event Study Estimates of the FFCRA Medicaid Continuity Effects on Children’s Insurance coverage | | | | | | |
| --- | --- | --- | --- | --- | --- | --- |
|  | Public coverage | Any private coverage | Employer-sponsored coverage | Privately purchased coverage | Any insurance coverage | Insurance gap |
| **EVENT STUDY DD ESTIMATES** |  |  |  |  |  |  |
| 2016🞨MC | 0.043 | -0.037 | -0.017 | -0.034* | 0.012 | -0.0063 |
|  | [-0.0013,0.088] | [-0.11,0.038] | [-0.082,0.048] | [-0.066,-0.0030] | [-0.016,0.039] | [-0.037,0.025] |
| 2017🞨MC | 0.080*** | -0.026 | -0.0076 | -0.0094 | 0.021 | -0.0067 |
|  | [0.036,0.12] | [-0.062,0.011] | [-0.036,0.021] | [-0.031,0.012] | [-0.0018,0.043] | [-0.040,0.027] |
| 2018🞨MC | 0.076* | -0.058 | -0.022 | -0.038* | 0.016 | -0.0026 |
|  | [0.0100,0.14] | [-0.13,0.016] | [-0.080,0.035] | [-0.069,-0.0070] | [-0.0025,0.035] | [-0.033,0.028] |
| 2019🞨MC | Ref. | Ref. | Ref. | Ref. | Ref. | Ref. |
|  |  |  |  |  |  |  |
| 2020🞨MC | 0.041* | -0.033 | -0.0014 | -0.035*** | 0.014 | -0.0097 |
|  | [0.000037,0.083] | [-0.083,0.017] | [-0.048,0.046] | [-0.053,-0.017] | [-0.0028,0.030] | [-0.029,0.0095] |
| 2021🞨MC | 0.047* | -0.026 | -0.006 | -0.018 | 0.011 | -0.016 |
|  | [0.0044,0.090] | [-0.061,0.0085] | [-0.041,0.029] | [-0.043,0.0071] | [-0.015,0.038] | [-0.046,0.015] |
| 2022🞨MC | 0.054** | -0.027 | -0.0027 | -0.026 | 0.011 | -0.0063 |
|  | [0.020,0.087] | [-0.078,0.024] | [-0.049,0.043] | [-0.057,0.0045] | [-0.028,0.050] | [-0.050,0.037] |
| 2023🞨MC | 0.027 | -0.0079 | 0.0026 | -0.024 | 0.0062 | -0.0028 |
|  | [-0.012,0.067] | [-0.059,0.043] | [-0.038,0.044] | [-0.050,0.0017] | [-0.021,0.033] | [-0.031,0.025] |
| **COVARIATES** |  |  |  |  |  |  |
| **Child’s Age** |  |  |  |  |  |  |
| Age 1 | Ref. | Ref. | Ref. | Ref. | Ref. | Ref. |
|  |  |  |  |  |  |  |
| Age 2 | -0.0027 | 0.01 | 0.006 | 0.0072 | 0.0088 | -0.025* |
|  | [-0.025,0.019] | [-0.018,0.038] | [-0.019,0.031] | [-0.0038,0.018] | [-0.0084,0.026] | [-0.047,-0.0026] |
| Age 3 | 0.0013 | -0.0078 | 0.00013 | -0.0018 | -0.0018 | -0.011 |
|  | [-0.022,0.024] | [-0.041,0.025] | [-0.035,0.035] | [-0.015,0.012] | [-0.024,0.020] | [-0.029,0.0082] |
| Age 4 | -0.011 | 0.027 | 0.024 | 0.0061 | 0.011 | -0.017 |
|  | [-0.046,0.023] | [-0.012,0.066] | [-0.0022,0.051] | [-0.013,0.025] | [-0.0055,0.026] | [-0.039,0.0051] |
| Age 5 | -0.021 | 0.030* | 0.032 | 0.0018 | 0.002 | -0.014 |
|  | [-0.048,0.0062] | [0.0011,0.058] | [-0.0055,0.069] | [-0.011,0.015] | [-0.018,0.022] | [-0.031,0.0025] |
| Age 6 | -0.030* | 0.023 | 0.02 | 0.023 | -0.0084 | -0.0016 |
|  | [-0.059,-0.0015] | [-0.017,0.063] | [-0.011,0.050] | [-0.0024,0.047] | [-0.029,0.013] | [-0.017,0.014] |
| Age 7 | -0.044* | 0.027** | 0.029** | 0.0013 | -0.012 | 0.0047 |
|  | [-0.082,-0.0054] | [0.0086,0.045] | [0.010,0.048] | [-0.011,0.014] | [-0.038,0.013] | [-0.020,0.030] |
| Age 8 | -0.013 | 0.014 | 0.015 | 0.0036 | 0.0068 | -0.016 |
|  | [-0.058,0.032] | [-0.023,0.051] | [-0.019,0.049] | [-0.0080,0.015] | [-0.0090,0.023] | [-0.034,0.0027] |
| Age 9 | -0.018 | 0.035* | 0.038*** | 0.0096 | 0.0094 | -0.025* |
|  | [-0.052,0.017] | [0.0042,0.066] | [0.017,0.059] | [-0.0047,0.024] | [-0.0080,0.027] | [-0.048,-0.0024] |
| Age 10 | -0.02 | 0.028 | 0.029 | 0.0058 | 0.006 | -0.014 |
|  | [-0.060,0.020] | [-0.016,0.071] | [-0.0094,0.067] | [-0.0098,0.021] | [-0.010,0.022] | [-0.033,0.0044] |
| Age 11 | -0.066*** | 0.062*** | 0.063*** | 0.0088 | -0.0029 | -0.015 |
|  | [-0.10,-0.032] | [0.035,0.088] | [0.037,0.088] | [-0.0074,0.025] | [-0.024,0.018] | [-0.042,0.012] |
| Age 12 | -0.075*** | 0.061*** | 0.059*** | 0.017* | -0.017 | -0.00077 |
|  | [-0.10,-0.048] | [0.036,0.086] | [0.030,0.088] | [0.0015,0.033] | [-0.044,0.011] | [-0.019,0.017] |
| Age 13 | -0.062*** | 0.055** | 0.043** | 0.024*** | -0.0086 | -0.012 |
|  | [-0.097,-0.026] | [0.023,0.087] | [0.016,0.070] | [0.014,0.034] | [-0.022,0.0053] | [-0.032,0.0092] |
| Age 14 | -0.093*** | 0.059** | 0.047** | 0.024*** | -0.032** | 0.014 |
|  | [-0.12,-0.065] | [0.021,0.097] | [0.014,0.079] | [0.013,0.035] | [-0.055,-0.0091] | [-0.0053,0.033] |
| Age 15 | -0.088*** | 0.077*** | 0.052** | 0.029*** | -0.016 | 0.0045 |
|  | [-0.12,-0.060] | [0.047,0.11] | [0.020,0.084] | [0.019,0.040] | [-0.039,0.0071] | [-0.015,0.024] |
| Age 16 | -0.12*** | 0.100*** | 0.073*** | 0.034*** | -0.020* | 0.014 |
|  | [-0.15,-0.085] | [0.065,0.13] | [0.034,0.11] | [0.022,0.046] | [-0.039,-0.00059] | [-0.0092,0.037] |
| Age 17 | -0.13*** | 0.11*** | 0.062*** | 0.031*** | -0.019 | 0.011 |
|  | [-0.18,-0.074] | [0.083,0.14] | [0.030,0.094] | [0.016,0.046] | [-0.050,0.012] | [-0.018,0.040] |
| **Child’s Sex** |  |  |  |  |  |  |
| Male | Ref. | Ref. | Ref. | Ref. | Ref. | Ref. |
|  |  |  |  |  |  |  |
| Female | -0.013* | 0.0029 | 0.0041 | -0.0037 | -0.0052 | 0.0083 |
|  | [-0.025,-0.0019] | [-0.0030,0.0088] | [-0.0013,0.0095] | [-0.0080,0.00064] | [-0.014,0.0040] | [-0.0021,0.019] |
| **Child’s race/ethnicity** |  |  |  |  |  |  |
| Non-Hispanic White | Ref. | Ref. | Ref. | Ref. | Ref. | Ref. |
|  |  |  |  |  |  |  |
| Non-Hispanic Black | 0.11*** | -0.085*** | -0.080*** | 0.00071 | 0.0038 | 0.004 |
|  | [0.084,0.13] | [-0.11,-0.062] | [-0.11,-0.053] | [-0.0096,0.011] | [-0.0084,0.016] | [-0.010,0.018] |
| Non-Hispanic Others Races | 0.062*** | -0.040** | -0.051*** | 0.016* | 0.0083 | -0.0024 |
|  | [0.030,0.094] | [-0.067,-0.013] | [-0.077,-0.024] | [0.0034,0.028] | [-0.011,0.028] | [-0.018,0.014] |
| Hispanic | 0.060*** | -0.054*** | -0.054*** | -0.0032 | -0.001 | 0.0068 |
|  | [0.036,0.083] | [-0.074,-0.034] | [-0.071,-0.037] | [-0.0083,0.0019] | [-0.013,0.011] | [-0.0063,0.020] |
| **Highest education in household** |  |  |  |  |  |  |
| Less than High School | Ref. | Ref. | Ref. | Ref. | Ref. | Ref. |
|  |  |  |  |  |  |  |
| High School | 0.042 | 0.055*** | 0.062*** | -0.018* | 0.083*** | -0.074** |
|  | [-0.0052,0.089] | [0.029,0.080] | [0.043,0.082] | [-0.036,-0.00078] | [0.037,0.13] | [-0.12,-0.030] |
| Some College | -0.0048 | 0.14*** | 0.14*** | -0.014 | 0.11*** | -0.091*** |
|  | [-0.055,0.046] | [0.11,0.17] | [0.12,0.16] | [-0.030,0.0018] | [0.058,0.16] | [-0.14,-0.046] |
| College and above | -0.18*** | 0.31*** | 0.29*** | 0.0035 | 0.12*** | -0.11*** |
|  | [-0.23,-0.13] | [0.27,0.35] | [0.26,0.32] | [-0.018,0.025] | [0.069,0.17] | [-0.16,-0.056] |
| **Number of kids in household** |  |  |  |  |  |  |
| 1 | Ref. | Ref. | Ref. | Ref. | Ref. | Ref. |
|  |  |  |  |  |  |  |
| 2 | -0.000049 | 0.017 | 0.025** | -0.010** | 0.0098** | -0.0085* |
|  | [-0.022,0.022] | [-0.00075,0.035] | [0.0098,0.041] | [-0.017,-0.0041] | [0.0033,0.016] | [-0.015,-0.0016] |
| 3 | 0.024*** | -0.015 | 0.002 | -0.018*** | 0.0017 | -0.0084 |
|  | [0.012,0.036] | [-0.031,0.00098] | [-0.011,0.015] | [-0.026,-0.0100] | [-0.0069,0.010] | [-0.022,0.0050] |
| 4 or More | 0.061*** | -0.056*** | -0.030** | -0.029*** | -0.016 | 0.003 |
|  | [0.040,0.082] | [-0.076,-0.036] | [-0.051,-0.0093] | [-0.037,-0.021] | [-0.033,0.00088] | [-0.014,0.020] |
| **Household income** |  |  |  |  |  |  |
| Below 100% FPL | Ref. | Ref. | Ref. | Ref. | Ref. | Ref. |
|  |  |  |  |  |  |  |
| 100-199% FPL | -0.051*** | 0.087*** | 0.080*** | 0.0062 | 0.016** | -0.013** |
|  | [-0.071,-0.031] | [0.069,0.10] | [0.065,0.095] | [-0.000011,0.012] | [0.0053,0.026] | [-0.022,-0.0039] |
| 200-300% FPL | -0.28*** | 0.30*** | 0.28*** | 0.018*** | 0.017* | -0.021** |
|  | [-0.30,-0.26] | [0.28,0.32] | [0.26,0.30] | [0.011,0.024] | [0.0040,0.029] | [-0.035,-0.0069] |
| **Parental Marital Status** |  |  |  |  |  |  |
| Not Married | Ref. | Ref. | Ref. | Ref. | Ref. | Ref. |
|  |  |  |  |  |  |  |
| Married | -0.10*** | 0.082*** | 0.063*** | 0.017*** | -0.0051 | 0.0025 |
|  | [-0.11,-0.092] | [0.070,0.094] | [0.051,0.075] | [0.0096,0.023] | [-0.014,0.0040] | [-0.0089,0.014] |
| **Household employment** |  |  |  |  |  |  |
| No employed | Ref. | Ref. | Ref. | Ref. | Ref. | Ref. |
|  |  |  |  |  |  |  |
| At least one employed | -0.17*** | 0.18*** | 0.18*** | 0.013*** | 0.0055 | -0.025** |
|  | [-0.18,-0.15] | [0.16,0.19] | [0.16,0.20] | [0.0057,0.020] | [-0.010,0.022] | [-0.042,-0.0083] |
| **Primary household language** |  |  |  |  |  |  |
| English | Ref. | Ref. | Ref. | Ref. | Ref. | Ref. |
|  |  |  |  |  |  |  |
| Spanish | 0.093*** | -0.13*** | -0.13*** | 0.0073 | -0.030** | 0.042** |
|  | [0.058,0.13] | [-0.15,-0.11] | [-0.15,-0.11] | [-0.012,0.026] | [-0.052,-0.0090] | [0.014,0.070] |
| Other | 0.011 | -0.10*** | -0.12*** | 0.024*** | -0.099*** | 0.097*** |
| **State-Level Covariates** | [-0.053,0.074] | [-0.14,-0.072] | [-0.15,-0.088] | [0.015,0.032] | [-0.15,-0.051] | [0.052,0.14] |
| State Unemployment Rate | -0.008 | -0.0025 | -0.0025 | -0.0045* | -0.0055* | 0.0087** |
|  | [-0.016,0.00049] | [-0.0073,0.0024] | [-0.0084,0.0034] | [-0.0081,-0.00095] | [-0.010,-0.00083] | [0.0030,0.014] |
| State Medicaid Expansion Status | 0.0038 | -0.00093 | -0.0025 | -0.0039 | 0.0034 | 0.001 |
|  | [-0.033,0.040] | [-0.020,0.019] | [-0.021,0.016] | [-0.016,0.0084] | [-0.012,0.019] | [-0.020,0.023] |
|  |  |  |  |  |  |  |
| **State Fixed Effects** | YES | YES | YES | YES | YES | YES |
| **Year Fixed Effects** | YES | YES | YES | YES | YES | YES |
| *Outcome Mean in 2019* | 0.52 | 0.46 | 0.39 | 0.064 | 0.91 | 0.12 |
| *N* | 124463 | 124463 | 124420 | 122901 | 125990 | 125380 |
| Notes: 95% confidence intervals are in brackets and derived from state-clustered standard errors. The sample includes children aged 1-17 years in families with income up to 300% federal poverty level. The estimates are obtained from a difference-in-difference event-study regression that estimates year-by-year differences in outcomes from 2016 to 2023 versus 2019 as the reference year between the treatment and control groups. All models adjust for age, sex, race/ethnicity, total number of children living in the household, highest educational level of parents/caregivers, marital status, any household employment, family income, state Medicaid expansion status, state unemployment rate, survey year, and state of residence. Coefficients for state and year fixed effects are omitted from this table for brevity. All models are weighed by the NSCH sampling weights to yield nationally representative estimates.  *p<0.05, **p<0.01, ***p<0.001; Abbreviation: FFCRA, Families First Coronavirus Response Act; NSCH, National Survey of Children’s Health; MC, States without 12-month continuous Medicaid Coverage before the FFCRA in 2019 (i.e., the treatment group indicator). | | | | | | |

| Appendix Figure 2. Confidence Bounds for the Estimated FFCRA Effects on Coverage Outcomes Accounting for Pre-trend Differences between Treatment and Control States |
| --- |
|  |
| Notes: This figure presents 95% confidence bounds that account for pre-trend differences between the treatment and control groups following the approach of Rambachan and Roth (2023) based on specifying smoothness restrictions on the extent to which the differential pre-trend changes over time. These are presented for the difference-in-differences estimates of the FFCRA effects that were statistically significant when assuming no pre-trend differences between the treatment and control groups. In each panel, the red bar (labeled “Original”) represents the 95% confidence interval of the original estimate assuming no pre-trend difference. The blue bars represent the 95% confidence intervals allowing for pre-trend differences. These intervals are constructed for different values of the smoothness parameter M, which indicates the period-to-period change in the slope of the pre-trend. M=0 assumes that the differential trend observed in the pre-treatment period continues linearly into the post-treatment period; larger values of M allow for non-linear deviations from the pre-trend.  Abbreviation: FFCRA, Families First Coronavirus Response Act |

| Appendix Figure 3. Descriptive Unadjusted Trends of Children’s Access to Care Outcomes between States with and without 12-Month Continuous Medicaid Coverage from 2016 to 2023 |
| --- |
|  |
| Notes: The sample includes children aged 1-17 years in families with income up to 300% of the federal poverty level. The statistics are all weighted by the NSCH sampling weights.  Abbreviation: FFCRA, Families First Coronavirus Response Act; NSCH, National Survey of Children’s Health |

| Appendix Table 3. Difference-in-Differences Event Study Estimates of the FFCRA Medicaid Continuity on Children’s Access to Care | | | | |
| --- | --- | --- | --- | --- |
|  | Usual place for sick care | Usual place for preventive care | Have a personal doctor | Unmet care needs |
| **EVENT STUDY DD ESTIMATES** |  |  |  |  |
| 2016🞨MC | 0.02 | -0.00055 | -0.025 | -0.0063 |
|  | [-0.012,0.052] | [-0.036,0.034] | [-0.067,0.017] | [-0.017,0.0042] |
| 2017🞨MC | -0.0052 | 0.0059 | -0.011 | -0.0054 |
|  | [-0.038,0.027] | [-0.021,0.032] | [-0.061,0.039] | [-0.024,0.013] |
| 2018🞨MC | 0.021 | 0.032 | 0.034 | -0.0092 |
|  | [-0.019,0.061] | [-0.015,0.080] | [-0.021,0.089] | [-0.021,0.0024] |
| 2019🞨MC | Ref. | Ref. | Ref. | Ref. |
|  |  |  |  |  |
| 2020🞨MC | -0.023 | -0.0086 | -0.0054 | -0.0077 |
|  | [-0.047,0.0012] | [-0.046,0.029] | [-0.037,0.026] | [-0.025,0.0099] |
| 2021🞨MC | 0.036* | 0.031 | 0.026 | -0.017** |
|  | [0.0045,0.068] | [-0.0047,0.066] | [-0.016,0.068] | [-0.028,-0.0067] |
| 2022🞨MC | 0.013 | 0.019 | 0.022 | -0.024** |
|  | [-0.015,0.041] | [-0.0065,0.044] | [-0.012,0.057] | [-0.038,-0.0096] |
| 2023🞨MC | 0.023 | 0.017 | -0.001 | 0.000084 |
|  | [-0.012,0.058] | [-0.017,0.051] | [-0.052,0.050] | [-0.017,0.017] |
| **COVARIATES** |  |  |  |  |
| **Child’s Age** |  |  |  |  |
| Age 1 | Ref. | Ref. | Ref. | Ref. |
|  |  |  |  |  |
| Age 2 | 0.042 | 0.015 | 0.033 | 0.00021 |
|  | [-0.0048,0.088] | [-0.011,0.041] | [-0.0040,0.070] | [-0.0072,0.0077] |
| Age 3 | 0.029 | -0.0064 | 0.03 | 0.0036 |
|  | [-0.011,0.069] | [-0.050,0.037] | [-0.0010,0.061] | [-0.0040,0.011] |
| Age 4 | 0.02 | 0.0087 | 0.027 | 0.015** |
|  | [-0.026,0.066] | [-0.012,0.029] | [-0.011,0.064] | [0.0057,0.024] |
| Age 5 | 0.008 | -0.0081 | -0.002 | 0.018* |
|  | [-0.044,0.060] | [-0.040,0.024] | [-0.042,0.038] | [0.0024,0.034] |
| Age 6 | 0.04 | 0.0041 | -0.0052 | 0.022*** |
|  | [-0.0015,0.082] | [-0.018,0.026] | [-0.028,0.018] | [0.011,0.033] |
| Age 7 | 0.041 | 0.0085 | 0.012 | 0.015** |
|  | [-0.012,0.095] | [-0.015,0.032] | [-0.019,0.043] | [0.0049,0.025] |
| Age 8 | 0.043 | 0.0075 | 0.034* | 0.030*** |
|  | [-0.0014,0.087] | [-0.017,0.033] | [0.0051,0.064] | [0.019,0.041] |
| Age 9 | 0.027 | 0.0098 | 0.018 | 0.018** |
|  | [-0.021,0.074] | [-0.015,0.035] | [-0.0082,0.044] | [0.0048,0.031] |
| Age 10 | 0.012 | -0.017 | -0.031 | 0.031*** |
|  | [-0.040,0.063] | [-0.044,0.010] | [-0.063,0.00054] | [0.017,0.045] |
| Age 11 | 0.022 | -0.014 | 0.02 | 0.030*** |
|  | [-0.0076,0.051] | [-0.044,0.015] | [-0.014,0.053] | [0.022,0.038] |
| Age 12 | -0.0026 | -0.011 | 0.021 | 0.025*** |
|  | [-0.042,0.037] | [-0.030,0.0071] | [-0.0047,0.047] | [0.011,0.039] |
| Age 13 | 0.019 | -0.0033 | 0.036** | 0.026*** |
|  | [-0.019,0.057] | [-0.023,0.017] | [0.010,0.061] | [0.018,0.033] |
| Age 14 | 0.014 | -0.013 | 0.0011 | 0.029*** |
|  | [-0.018,0.045] | [-0.033,0.0070] | [-0.026,0.028] | [0.017,0.040] |
| Age 15 | -0.023 | -0.032* | 0.009 | 0.045*** |
|  | [-0.061,0.016] | [-0.064,-0.00029] | [-0.018,0.036] | [0.033,0.057] |
| Age 16 | -0.0004 | -0.039** | -0.029* | 0.045*** |
|  | [-0.057,0.056] | [-0.067,-0.012] | [-0.057,-0.00047] | [0.027,0.064] |
| Age 17 | 0.0076 | -0.026* | -0.034 | 0.049*** |
|  | [-0.052,0.067] | [-0.051,-0.00075] | [-0.074,0.0052] | [0.035,0.062] |
| **Child’s Sex** |  |  |  |  |
| Male | Ref. | Ref. | Ref. | Ref. |
|  |  |  |  |  |
| Female | -0.0073 | -0.00054 | -0.0073 | 0.00072 |
|  | [-0.022,0.0078] | [-0.0070,0.0059] | [-0.024,0.0097] | [-0.0056,0.0071] |
| **Child’s race/ethnicity** |  |  |  |  |
| Non-Hispanic White | Ref. | Ref. | Ref. | Ref. |
|  |  |  |  |  |
| Non-Hispanic Black | -0.092*** | -0.031*** | -0.074*** | -0.0011 |
|  | [-0.11,-0.076] | [-0.045,-0.016] | [-0.089,-0.058] | [-0.012,0.0095] |
| Non-Hispanic Others Races | -0.078*** | -0.034** | -0.061*** | -0.0029 |
|  | [-0.098,-0.059] | [-0.054,-0.014] | [-0.072,-0.051] | [-0.010,0.0046] |
| Hispanic | -0.065*** | -0.023** | -0.078*** | -0.00048 |
|  | [-0.084,-0.046] | [-0.037,-0.0077] | [-0.11,-0.049] | [-0.0082,0.0072] |
| **Highest education in household** |  |  |  |  |
| Less than High School | Ref. | Ref. | Ref. | Ref. |
|  |  |  |  |  |
| High School | 0.029** | 0.063*** | 0.056*** | -0.0045 |
|  | [0.0095,0.049] | [0.037,0.090] | [0.037,0.075] | [-0.014,0.0052] |
| Some College | 0.10*** | 0.11*** | 0.12*** | 0.0013 |
|  | [0.084,0.12] | [0.078,0.13] | [0.10,0.14] | [-0.013,0.016] |
| College and above | 0.15*** | 0.13*** | 0.17*** | -0.0038 |
|  | [0.13,0.18] | [0.10,0.15] | [0.15,0.20] | [-0.018,0.011] |
| **Number of kids in household** |  |  |  |  |
| 1 | Ref. | Ref. | Ref. | Ref. |
|  |  |  |  |  |
| 2 | 0.011 | 0.0018 | 0.0097 | -0.0058 |
|  | [-0.0022,0.025] | [-0.0083,0.012] | [-0.0038,0.023] | [-0.012,0.00027] |
| 3 | 0.0083 | 0.00016 | -0.0033 | -0.01 |
|  | [-0.0046,0.021] | [-0.012,0.012] | [-0.019,0.012] | [-0.022,0.0018] |
| 4 or More | -0.0027 | -0.019 | -0.012 | -0.0043 |
|  | [-0.024,0.019] | [-0.038,0.000051] | [-0.032,0.0069] | [-0.017,0.0082] |
| **Household income** |  |  |  |  |
| Below 100% FPL | Ref. | Ref. | Ref. | Ref. |
|  |  |  |  |  |
| 100-199% FPL | 0.048*** | 0.025*** | 0.048*** | 0.000044 |
|  | [0.029,0.067] | [0.015,0.036] | [0.035,0.061] | [-0.0042,0.0042] |
| 200-300% FPL | 0.062*** | 0.028*** | 0.053*** | -0.0093** |
|  | [0.039,0.085] | [0.015,0.041] | [0.037,0.068] | [-0.015,-0.0035] |
| **Parental Marital Status** |  |  |  |  |
| Not Married | Ref. | Ref. | Ref. | Ref. |
|  |  |  |  |  |
| Married | 0.0055 | -0.0094 | 0.015* | -0.0075 |
|  | [-0.0021,0.013] | [-0.022,0.0032] | [0.000052,0.029] | [-0.022,0.0074] |
| **Household employment** |  |  |  |  |
| No employed | Ref. | Ref. | Ref. | Ref. |
|  |  |  |  |  |
| At least one employed | 0.021 | 0.025 | -0.01 | -0.022** |
|  | [-0.0028,0.046] | [-0.015,0.064] | [-0.033,0.012] | [-0.036,-0.0074] |
| **Primary household language** |  |  |  |  |
| English | Ref. | Ref. | Ref. | Ref. |
|  |  |  |  |  |
| Spanish | -0.060*** | -0.062*** | -0.023 | 0.0024 |
|  | [-0.080,-0.040] | [-0.074,-0.050] | [-0.053,0.0069] | [-0.0082,0.013] |
| Other | -0.12*** | -0.18*** | -0.062*** | 0.0025 |
|  | [-0.15,-0.081] | [-0.21,-0.14] | [-0.087,-0.038] | [-0.013,0.018] |
| **State-Level Covariates** |  |  |  |  |
| State Unemployment Rate | -0.0012 | -0.0066* | -0.0076* | 0.0045*** |
|  | [-0.0075,0.0051] | [-0.012,-0.00080] | [-0.014,-0.0011] | [0.0024,0.0066] |
| State Medicaid Expansion Status | 0.026* | 0.0069 | -0.0024 | 0.0029 |
|  | [0.0039,0.049] | [-0.0087,0.022] | [-0.022,0.017] | [-0.0062,0.012] |
|  |  |  |  |  |
| **State Fixed Effects** | YES | YES | YES | YES |
| **Year Fixed Effects** | YES | YES | YES | YES |
| *Outcome Mean in 2019* | 0.73 | 0.88 | 0.66 | 0.035 |
| *N* | 125902 | 125520 | 125809 | 125989 |
| Notes: 95% confidence intervals are in brackets and derived from state-clustered standard errors. The sample includes children aged 1-17 years in families with income up to 300% federal poverty level. The estimates are obtained from a difference-in-difference event-study regression that estimates year-by-year differences in outcomes from 2016 to 2023 versus 2019 as the reference year between the treatment and control groups. All models adjust for age, sex, race/ethnicity, total number of children living in the household, highest educational level of parents/caregivers, marital status, any household employment, family income, state Medicaid expansion status, state unemployment rate, survey year, and state of residence. Coefficients for state and year fixed effects are omitted from this table for brevity. All models are weighed by the NSCH sampling weights to yield nationally representative estimates.  *p<0.05, **p<0.01, ***p<0.001; Abbreviation: FFCRA, Families First Coronavirus Response Act; NSCH, National Survey of Children’s Health; MC, States without 12-month continuous Medicaid Coverage before the FFCRA in 2019 (i.e., the treatment group indicator). | | | | |

| Appendix Figure 4. Confidence Bounds for the Estimated FFCRA Effects on Access to Care Outcomes Accounting for Pre-trend Differences between Treatment and Control States |
| --- |
|  |
| Notes: This figure presents 95% confidence bounds that account for pre-trend differences between the treatment and control groups following the approach of Rambachan and Roth (2023) based on specifying smoothness restrictions on the extent to which the differential pre-trend changes over time. These are presented for the difference-in-differences estimates of the FFCRA effects that were statistically significant when assuming no pre-trend differences between the treatment and control groups. In each panel, the red bar (labeled “Original”) represents the 95% confidence interval of the original estimate assuming no pre-trend difference. The blue bars represent the 95% confidence intervals allowing for pre-trend differences. These intervals are constructed for different values of the smoothness parameter M, which indicates the period-to-period change in the slope of the pre-trend. M=0 assumes that the differential trend observed in the pre-treatment period continues linearly into the post-treatment period; larger values of M allow for non-linear deviations from the pre-trend.  Abbreviation: FFCRA, Families First Coronavirus Response Act |

| Appendix Figure 5. Descriptive Trends of Children’s Use of Health Servces by Type between States with and without 12-Month Continuous Medicaid Coverage from 2016 to 2023 |
| --- |
|  |
| Notes: The sample includes children aged 1-17 years in families with income up to 300% federal poverty level. The statistics are all weighted by the NSCH sampling weights.  Abbreviation: FFCRA, Families First Coronavirus Response Act; NSCH, National Survey of Children’s Health |

| Appendix Table 4. Difference-in-Differences Event Study Estimates of the FFCRA Medicaid Continuity Effects on Children’s Use of Health Servces by Type | | | | | | |
| --- | --- | --- | --- | --- | --- | --- |
|  | Any Medical Visits | Any preventive Check-up | Any mental visits | Any specialist visits | Any ED visits | Any hospital stay |
| **EVENT STUDY DD ESTIMATES** |  |  |  |  |  |  |
| 2016🞨MC | 0.00023 | 0.017 | 0.0012 | -0.019 | 0.0087 | N.A. |
|  | [-0.049,0.049] | [-0.031,0.064] | [-0.023,0.025] | [-0.050,0.012] | [-0.026,0.043] |  |
| 2017🞨MC | 0.0011 | 0.023 | -0.015 | -0.042** | 0.019 | N.A. |
|  | [-0.035,0.037] | [-0.0099,0.056] | [-0.040,0.010] | [-0.067,-0.018] | [-0.019,0.057] |  |
| 2018🞨MC | 0.012 | 0.03 | -0.0014 | -0.019 | 0.026 | 0.01 |
|  | [-0.037,0.061] | [-0.012,0.071] | [-0.027,0.024] | [-0.043,0.0042] | [-0.0069,0.058] | [-0.0097,0.030] |
| 2019🞨MC | Ref. | Ref. | Ref. | Ref. | Ref. | Ref. |
|  |  |  |  |  |  |  |
| 2020🞨MC | 0.017 | 0.04 | -0.017 | -0.019 | 0.018 | 0.0058 |
|  | [-0.033,0.067] | [-0.016,0.097] | [-0.045,0.011] | [-0.039,0.00093] | [-0.018,0.054] | [-0.012,0.024] |
| 2021🞨MC | 0.0052 | 0.023 | -0.02 | -0.015 | 0.026 | 0.0095 |
|  | [-0.028,0.039] | [-0.019,0.066] | [-0.043,0.0034] | [-0.044,0.015] | [-0.011,0.063] | [-0.0043,0.023] |
| 2022🞨MC | 0.021 | 0.029 | -0.0069 | -0.012 | 0.018 | 0.014 |
|  | [-0.037,0.078] | [-0.018,0.076] | [-0.028,0.014] | [-0.040,0.015] | [-0.014,0.050] | [-0.0047,0.033] |
| 2023🞨MC | 0.0078 | 0.022 | -0.014 | -0.025 | 0.014 | 0.0061 |
|  | [-0.034,0.049] | [-0.015,0.058] | [-0.037,0.0099] | [-0.050,0.00039] | [-0.020,0.049] | [-0.014,0.026] |
| **COVARIATES** |  |  |  |  |  |  |
| **Child’s Age** |  |  |  |  |  |  |
| Age 1 | Ref. | Ref. | Ref. | Ref. | Ref. | Ref. |
|  |  |  |  |  |  |  |
| Age 2 | -0.0072 | -0.014 | 0.0051 | -0.014 | -0.028 | -0.036** |
|  | [-0.026,0.012] | [-0.038,0.0089] | [-0.0025,0.013] | [-0.033,0.0052] | [-0.076,0.020] | [-0.057,-0.015] |
| Age 3 | -0.059** | -0.077*** | 0.0088* | -0.013 | -0.10*** | -0.056*** |
|  | [-0.099,-0.019] | [-0.12,-0.035] | [0.0022,0.015] | [-0.027,0.0021] | [-0.14,-0.063] | [-0.079,-0.034] |
| Age 4 | -0.030* | -0.048*** | 0.021** | -0.016 | -0.10*** | -0.058*** |
|  | [-0.052,-0.0073] | [-0.073,-0.023] | [0.0071,0.035] | [-0.043,0.011] | [-0.14,-0.066] | [-0.075,-0.041] |
| Age 5 | -0.055*** | -0.071*** | 0.041*** | -0.021** | -0.12*** | -0.056*** |
|  | [-0.078,-0.031] | [-0.099,-0.043] | [0.033,0.048] | [-0.037,-0.0063] | [-0.17,-0.072] | [-0.077,-0.034] |
| Age 6 | -0.084*** | -0.100*** | 0.060*** | -0.0043 | -0.14*** | -0.061*** |
|  | [-0.11,-0.063] | [-0.12,-0.075] | [0.054,0.067] | [-0.024,0.016] | [-0.18,-0.094] | [-0.079,-0.044] |
| Age 7 | -0.099*** | -0.14*** | 0.068*** | -0.012 | -0.16*** | -0.055*** |
|  | [-0.13,-0.066] | [-0.19,-0.087] | [0.049,0.087] | [-0.038,0.015] | [-0.21,-0.12] | [-0.075,-0.036] |
| Age 8 | -0.10*** | -0.13*** | 0.088*** | -0.013 | -0.16*** | -0.060*** |
|  | [-0.12,-0.077] | [-0.15,-0.11] | [0.067,0.11] | [-0.028,0.0021] | [-0.20,-0.12] | [-0.077,-0.043] |
| Age 9 | -0.13*** | -0.16*** | 0.11*** | -0.0036 | -0.16*** | -0.058*** |
|  | [-0.17,-0.089] | [-0.21,-0.12] | [0.082,0.14] | [-0.024,0.017] | [-0.21,-0.12] | [-0.079,-0.038] |
| Age 10 | -0.13*** | -0.17*** | 0.10*** | -0.023*** | -0.18*** | -0.058*** |
|  | [-0.18,-0.088] | [-0.22,-0.11] | [0.077,0.12] | [-0.036,-0.0100] | [-0.23,-0.13] | [-0.077,-0.039] |
| Age 11 | -0.12*** | -0.15*** | 0.095*** | 0.0031 | -0.19*** | -0.061*** |
|  | [-0.16,-0.074] | [-0.20,-0.096] | [0.072,0.12] | [-0.0067,0.013] | [-0.24,-0.13] | [-0.082,-0.040] |
| Age 12 | -0.13*** | -0.16*** | 0.12*** | 0.0035 | -0.20*** | -0.063*** |
|  | [-0.17,-0.093] | [-0.20,-0.13] | [0.099,0.14] | [-0.022,0.029] | [-0.24,-0.16] | [-0.083,-0.044] |
| Age 13 | -0.14*** | -0.16*** | 0.13*** | 0.016 | -0.17*** | -0.052*** |
|  | [-0.17,-0.11] | [-0.19,-0.14] | [0.11,0.14] | [-0.013,0.045] | [-0.22,-0.11] | [-0.071,-0.033] |
| Age 14 | -0.17*** | -0.20*** | 0.13*** | 0.0056 | -0.19*** | -0.047*** |
|  | [-0.21,-0.13] | [-0.24,-0.17] | [0.11,0.15] | [-0.010,0.021] | [-0.23,-0.16] | [-0.065,-0.030] |
| Age 15 | -0.16*** | -0.19*** | 0.14*** | 0.032*** | -0.19*** | -0.053*** |
|  | [-0.19,-0.14] | [-0.22,-0.16] | [0.12,0.16] | [0.017,0.047] | [-0.23,-0.15] | [-0.069,-0.037] |
| Age 16 | -0.15*** | -0.20*** | 0.15*** | 0.025** | -0.17*** | -0.053*** |
|  | [-0.18,-0.12] | [-0.22,-0.17] | [0.12,0.18] | [0.0074,0.044] | [-0.22,-0.12] | [-0.074,-0.032] |
| Age 17 | -0.18*** | -0.21*** | 0.14*** | 0.034*** | -0.18*** | -0.040*** |
|  | [-0.22,-0.14] | [-0.25,-0.18] | [0.12,0.16] | [0.016,0.051] | [-0.22,-0.14] | [-0.056,-0.025] |
| **Child’s Sex** |  |  |  |  |  |  |
| Male | Ref. | Ref. | Ref. | Ref. | Ref. | Ref. |
|  |  |  |  |  |  |  |
| Female | 0.003 | 0.0027 | -0.0019 | -0.0096** | -0.016*** | -0.0032 |
|  | [-0.011,0.016] | [-0.012,0.017] | [-0.0091,0.0053] | [-0.016,-0.0031] | [-0.024,-0.0075] | [-0.0081,0.0017] |
| **Child’s race/ethnicity** |  |  |  |  |  |  |
| Non-Hispanic White | Ref. | Ref. | Ref. | Ref. | Ref. | Ref. |
|  |  |  |  |  |  |  |
| Non-Hispanic Black | -0.017** | -0.0027 | -0.041*** | -0.048*** | 0.062*** | 0.0069 |
|  | [-0.029,-0.0048] | [-0.018,0.013] | [-0.050,-0.031] | [-0.058,-0.038] | [0.046,0.078] | [-0.00044,0.014] |
| Non-Hispanic Others Races | -0.037** | -0.031** | -0.026** | -0.031*** | 0.0066 | 0.0044 |
|  | [-0.060,-0.015] | [-0.053,-0.0091] | [-0.042,-0.010] | [-0.048,-0.013] | [-0.0053,0.019] | [-0.0046,0.013] |
| Hispanic | -0.017* | -0.0047 | -0.015* | -0.023*** | 0.026** | 0.010* |
|  | [-0.034,-0.00012] | [-0.024,0.014] | [-0.029,-0.00070] | [-0.030,-0.016] | [0.010,0.042] | [0.0022,0.018] |
| **Highest education in household** |  |  |  |  |  |  |
| Less than High School | Ref. | Ref. | Ref. | Ref. | Ref. | Ref. |
|  |  |  |  |  |  |  |
| High School | 0.076*** | 0.081*** | 0.0095* | 0.023*** | 0.0018 | 0.011* |
|  | [0.036,0.12] | [0.044,0.12] | [0.00065,0.018] | [0.010,0.036] | [-0.027,0.030] | [0.0022,0.020] |
| Some College | 0.12*** | 0.13*** | 0.020** | 0.041*** | 0.01 | 0.012 |
|  | [0.079,0.16] | [0.089,0.17] | [0.0082,0.032] | [0.030,0.052] | [-0.026,0.046] | [-0.0017,0.027] |
| College and above | 0.16*** | 0.17*** | 0.021** | 0.065*** | -0.048*** | 0.010* |
|  | [0.12,0.20] | [0.13,0.21] | [0.0083,0.035] | [0.054,0.077] | [-0.074,-0.022] | [0.0017,0.019] |
| **Number of kids in household** |  |  |  |  |  |  |
| 1 | Ref. | Ref. | Ref. | Ref. | Ref. | Ref. |
|  |  |  |  |  |  |  |
| 2 | 0.0041 | 0.012* | -0.0006 | 0.0011 | -0.016** | -0.0013 |
|  | [-0.0046,0.013] | [0.0013,0.023] | [-0.0081,0.0069] | [-0.011,0.014] | [-0.026,-0.0060] | [-0.0061,0.0035] |
| 3 | -0.012* | -0.0089 | -0.0061 | -0.0098* | -0.015* | -0.0034 |
|  | [-0.023,-0.0022] | [-0.021,0.0027] | [-0.017,0.0050] | [-0.018,-0.0017] | [-0.028,-0.0016] | [-0.0083,0.0015] |
| 4 or More | -0.031** | -0.025* | -0.0018 | -0.0068 | -0.030* | 0.0017 |
|  | [-0.049,-0.012] | [-0.048,-0.0035] | [-0.015,0.012] | [-0.021,0.0071] | [-0.055,-0.0043] | [-0.0075,0.011] |
| **Household income** |  |  |  |  |  |  |
| Below 100% FPL | Ref. | Ref. | Ref. | Ref. | Ref. | Ref. |
|  |  |  |  |  |  |  |
| 100-199% FPL | 0.038*** | 0.035*** | 0.0038 | 0.0046 | -0.035*** | -0.0029 |
|  | [0.026,0.050] | [0.022,0.048] | [-0.0034,0.011] | [-0.0033,0.013] | [-0.046,-0.023] | [-0.0098,0.0041] |
| 200-300% FPL | 0.040*** | 0.038*** | -0.0052 | 0.0052 | -0.061*** | -0.0079** |
|  | [0.030,0.050] | [0.027,0.048] | [-0.012,0.0015] | [-0.0013,0.012] | [-0.073,-0.049] | [-0.013,-0.0025] |
| **Parental Marital Status** |  |  |  |  |  |  |
| Not Married | Ref. | Ref. | Ref. | Ref. | Ref. | Ref. |
|  |  |  |  |  |  |  |
| Married | -0.016** | -0.01 | -0.055*** | -0.011* | -0.043*** | -0.0071* |
|  | [-0.025,-0.0063] | [-0.021,0.00045] | [-0.066,-0.045] | [-0.019,-0.0025] | [-0.053,-0.034] | [-0.013,-0.00090] |
| **Household employment** |  |  |  |  |  |  |
| No employed | Ref. | Ref. | Ref. | Ref. | Ref. | Ref. |
|  |  |  |  |  |  |  |
| At least one employed | -0.007 | -0.0049 | -0.043*** | -0.015 | -0.024* | -0.012** |
|  | [-0.029,0.015] | [-0.036,0.026] | [-0.065,-0.021] | [-0.037,0.0068] | [-0.043,-0.0040] | [-0.021,-0.0041] |
| **Primary household language** |  |  |  |  |  |  |
| English | Ref. | Ref. | Ref. | Ref. | Ref. | Ref. |
|  |  |  |  |  |  |  |
| Spanish | -0.039*** | -0.042*** | -0.032*** | -0.0033 | -0.019* | -0.0079 |
|  | [-0.055,-0.023] | [-0.053,-0.031] | [-0.046,-0.018] | [-0.014,0.0075] | [-0.035,-0.0035] | [-0.021,0.0057] |
| Other | -0.11*** | -0.11*** | -0.056*** | -0.017 | -0.032** | 0.0015 |
|  | [-0.15,-0.074] | [-0.14,-0.080] | [-0.063,-0.048] | [-0.041,0.0073] | [-0.053,-0.010] | [-0.0097,0.013] |
| **State-Level Covariates** |  |  |  |  |  |  |
| State Unemployment Rate | -0.0039 | -0.005 | 0.0019 | 0.0027 | -0.0024 | 0.00031 |
|  | [-0.012,0.0039] | [-0.015,0.0051] | [-0.0035,0.0074] | [-0.0011,0.0065] | [-0.0096,0.0048] | [-0.0022,0.0028] |
| State Medicaid Expansion Status | -0.01 | -0.0019 | 0.0015 | -0.023* | 0.0013 | 0.0011 |
|  | [-0.031,0.010] | [-0.017,0.014] | [-0.011,0.014] | [-0.041,-0.0056] | [-0.024,0.026] | [-0.014,0.016] |
|  |  |  |  |  |  |  |
| **State Fixed Effects** | YES | YES | YES | YES | YES | YES |
| **Year Fixed Effects** | YES | YES | YES | YES | YES | YES |
| *Outcome Mean in 2019* | 0.85 | 0.8 | 0.097 | 0.12 | 0.21 | 0.036 |
| *N* | 126247 | 125560 | 125847 | 125633 | 125760 | 100229 |
| Notes: 95% confidence intervals are in brackets and derived from state-clustered standard errors. The sample includes children aged 1-17 years in families with income up to 300% federal poverty level. The estimates are obtained from a difference-in-difference event-study regression that estimates year-by-year differences in outcomes from 2016 to 2023 versus 2019 as the reference year between the treatment and control groups. The 2016 and 2017 estimates for any hospital stay have been suppressed because no data are available for these years. All models adjust for age, sex, race/ethnicity, total number of children living in the household, highest educational level of parents/caregivers, marital status, any household employment, family income, state Medicaid expansion status, state unemployment rate, survey year, and state of residence. Coefficients for state and year fixed effects are omitted from this table for brevity. All models are weighed by the NSCH sampling weights to yield nationally representative estimates.  *p<0.05, **p<0.01, ***p<0.001; Abbreviation: FFCRA, Families First Coronavirus Response Act; NSCH, National Survey of Children’s Health; MC, States without 12-month continuous Medicaid Coverage before the FFCRA in 2019 (i.e., the treatment group indicator). | | | | | | |

| Appendix Figure 6. Descriptive Unadjusted Trends of Children’s Health Status as Rated by Parents/Caregivers between States with and without 12-Month Continuous Medicaid Coverage from 2016 to 2023 |
| --- |
|  |
| Notes: The sample includes children aged 1-17 years in families with income up to 300% federal poverty level. The statistics are all weighted by the NSCH sampling weights. |

| Appendix Table 5. Difference-in-Differences Event Study Estimates of the FFCRA Medicaid Continuity Effects on Children’s Health Status as Rated by Parents/Caregivers | | |
| --- | --- | --- |
|  | Any Medical Visits | Any preventive Check-up |
| **EVENT STUDY DD ESTIMATES** |  |  |
| 2016🞨MC | 0.015 | 0.00064 |
|  | [-0.0035,0.034] | [-0.011,0.012] |
| 2017🞨MC | 0.02 | 0.00088 |
|  | [-0.025,0.066] | [-0.0083,0.010] |
| 2018🞨MC | 0.018 | 0.005 |
|  | [-0.0039,0.039] | [-0.0079,0.018] |
| 2019🞨MC | Ref. | Ref. |
|  |  |  |
| 2020🞨MC | 0.025* | -0.0065 |
|  | [0.0043,0.045] | [-0.020,0.0068] |
| 2021🞨MC | 0.038* | -0.0028 |
|  | [0.0072,0.068] | [-0.020,0.015] |
| 2022🞨MC | 0.0041 | -0.0032 |
|  | [-0.020,0.029] | [-0.011,0.0049] |
| 2023🞨MC | 0.027* | 0.0011 |
|  | [0.0042,0.050] | [-0.0066,0.0088] |
| **COVARIATES** |  |  |
| **Child’s Age** |  |  |
| Age 1 | Ref. | Ref. |
|  |  |  |
| Age 2 | -0.006 | 0.0019 |
|  | [-0.027,0.015] | [-0.0035,0.0074] |
| Age 3 | -0.014 | 0.0083 |
|  | [-0.043,0.015] | [-0.00091,0.017] |
| Age 4 | -0.029** | 0.0088* |
|  | [-0.048,-0.011] | [0.00036,0.017] |
| Age 5 | -0.025*** | 0.0056* |
|  | [-0.039,-0.011] | [0.00059,0.011] |
| Age 6 | -0.027* | 0.0076* |
|  | [-0.050,-0.0038] | [0.00076,0.014] |
| Age 7 | -0.041*** | 0.0042 |
|  | [-0.053,-0.029] | [-0.0017,0.010] |
| Age 8 | -0.042*** | 0.012*** |
|  | [-0.055,-0.030] | [0.0052,0.018] |
| Age 9 | -0.044*** | 0.016** |
|  | [-0.060,-0.028] | [0.0057,0.026] |
| Age 10 | -0.065*** | 0.014*** |
|  | [-0.083,-0.046] | [0.0071,0.020] |
| Age 11 | -0.062*** | 0.0031 |
|  | [-0.080,-0.045] | [-0.0015,0.0076] |
| Age 12 | -0.078*** | 0.011* |
|  | [-0.092,-0.064] | [0.0027,0.020] |
| Age 13 | -0.075*** | 0.0072* |
|  | [-0.093,-0.057] | [0.00038,0.014] |
| Age 14 | -0.080*** | 0.015*** |
|  | [-0.098,-0.062] | [0.0062,0.023] |
| Age 15 | -0.086*** | 0.012** |
|  | [-0.11,-0.066] | [0.0033,0.020] |
| Age 16 | -0.082*** | 0.018 |
|  | [-0.10,-0.060] | [-0.0018,0.038] |
| Age 17 | -0.097*** | 0.034** |
|  | [-0.11,-0.080] | [0.014,0.054] |
| **Child’s Sex** |  |  |
| Male | Ref. | Ref. |
|  |  |  |
| Female | 0.0085 | 0.0025 |
|  | [-0.0041,0.021] | [-0.0012,0.0063] |
| **Child’s race/ethnicity** |  |  |
| Non-Hispanic White | Ref. | Ref. |
|  |  |  |
| Non-Hispanic Black | -0.041*** | 0.010** |
|  | [-0.058,-0.025] | [0.0030,0.017] |
| Non-Hispanic Others Races | -0.016* | 0.0028 |
|  | [-0.029,-0.0031] | [-0.00093,0.0066] |
| Hispanic | -0.016 | 0.0065** |
|  | [-0.032,0.0010] | [0.0023,0.011] |
| **Highest education in household** |  |  |
| Less than High School | Ref. | Ref. |
|  |  |  |
| High School | 0.025*** | -0.0077* |
|  | [0.012,0.039] | [-0.014,-0.0018] |
| Some College | 0.042*** | -0.0092*** |
|  | [0.028,0.057] | [-0.014,-0.0043] |
| College and above | 0.063*** | -0.013*** |
|  | [0.049,0.078] | [-0.018,-0.0075] |
| **Number of kids in household** |  |  |
| 1 | Ref. | Ref. |
|  |  |  |
| 2 | 0.0044 | 0.0038 |
|  | [-0.013,0.022] | [-0.0022,0.0098] |
| 3 | 0.0011 | 0.0044 |
|  | [-0.020,0.022] | [-0.0037,0.012] |
| 4 or More | 0.0062 | 0.009 |
|  | [-0.017,0.030] | [-0.0039,0.022] |
| **Household income** |  |  |
| Below 100% FPL | Ref. | Ref. |
|  |  |  |
| 100-199% FPL | 0.035*** | -0.0080*** |
|  | [0.021,0.050] | [-0.011,-0.0048] |
| 200-300% FPL | 0.052*** | -0.0098*** |
|  | [0.041,0.062] | [-0.014,-0.0052] |
| **Parental Marital Status** |  |  |
| Not Married | Ref. | Ref. |
|  |  |  |
| Married | 0.026*** | -0.0075*** |
|  | [0.015,0.037] | [-0.012,-0.0034] |
| **Household employment** |  |  |
| No employed | Ref. | Ref. |
|  |  |  |
| At least one employed | 0.040** | -0.0032 |
|  | [0.014,0.066] | [-0.013,0.0062] |
| **Primary household language** |  |  |
| English | Ref. | Ref. |
|  |  |  |
| Spanish | -0.041*** | 0.00049 |
|  | [-0.049,-0.032] | [-0.0058,0.0068] |
| Other | -0.039** | -0.0027 |
|  | [-0.064,-0.014] | [-0.010,0.0048] |
| **State-Level Covariates** |  |  |
| State Unemployment Rate | -0.0021 | 0.00019 |
|  | [-0.0074,0.0033] | [-0.0026,0.0030] |
| State Medicaid Expansion Status | -0.0077 | -0.0034 |
|  | [-0.028,0.013] | [-0.0084,0.0016] |
|  |  |  |
| **State Fixed Effects** | YES | YES |
| **Year Fixed Effects** | YES | YES |
| *Dep. Mean in 2019* | 0.87 | 0.022 |
| *N* | 126117 | 126117 |
| Notes: 95% confidence intervals are in brackets and derived from state-clustered standard errors. The sample includes children aged 1-17 years in families with income up to 300% federal poverty level. The estimates are obtained from a difference-in-difference event-study regression that estimates year-by-year differences in outcomes from 2016 to 2023 versus 2019 as the reference year between the treatment and control groups. All models adjust for age, sex, race/ethnicity, total number of children living in the household, highest educational level of parents/caregivers, marital status, any household employment, family income, state Medicaid expansion status, state unemployment rate, survey year, and state of residence. All models are weighed by the NSCH sampling weights to yield nationally representative estimates.  *p<0.05, **p<0.01, ***p<0.001; Abbreviation: FFCRA, Families First Coronavirus Response Act; NSCH, National Survey of Children’s Health; MC, States without 12-month continuous Medicaid Coverage before the FFCRA in 2019 (i.e., the treatment group indicator). | | |

| Appendix Figure 7. Confidence Bounds for the Estimated FFCRA Effects on Excellent/Very Good Health Outcome Accounting for Pre-trend Differences between Treatment and Control States |
| --- |
|  |
| Notes: This figure presents 95% confidence bounds that account for pre-trend differences between the treatment and control groups following the approach of Rambachan and Roth (2023) based on specifying smoothness restrictions on the extent to which the differential pre-trend changes over time. These are presented for the difference-in-differences estimates of the FFCRA effects that were statistically significant when assuming no pre-trend differences between the treatment and control groups. In each panel, the red bar (labeled “Original”) represents the 95% confidence interval of the original estimate assuming no pre-trend difference. The blue bars represent the 95% confidence intervals allowing for pre-trend differences. These intervals are constructed for different values of the smoothness parameter M, which indicates the period-to-period change in the slope of the pre-trend. M=0 assumes that the differential trend observed in the pre-treatment period continues linearly into the post-treatment period; larger values of M allow for non-linear deviations from the pre-trend.  Abbreviation: FFCRA, Families First Coronavirus Response Act |

| Appendix Table 6. Difference-in-Differences Event Study Estimates of the Federal Medicaid Continuous Coverage Requirement Effects on Children’s Health Insurance Coverage using Alternate Samples | | | | | | |
| --- | --- | --- | --- | --- | --- | --- |
|  | Medicaid coverage | Any private coverage | Employer-Sponsored coverage | Privately purchased coverage | Any insurance coverage | Insurance gap |
| ***Panel A: Medicaid Eligible Sample*** | | | | | | |
| 2016🞨MC | -0.022 | 0.0026 | 0.023 | -0.032** | 0.014 | -0.0077 |
|  | [-0.090,0.047] | [-0.057,0.062] | [-0.033,0.079] | [-0.053,-0.012] | [-0.020,0.048] | [-0.050,0.035] |
| 2017🞨MC | 0.043 | -0.02 | -0.013 | 0.0031 | 0.0057 | 0.004 |
|  | [-0.0075,0.094] | [-0.063,0.023] | [-0.060,0.034] | [-0.019,0.025] | [-0.032,0.044] | [-0.059,0.067] |
| 2018🞨MC | 0.033 | -0.026 | 0.0038 | -0.025** | 0.027 | -0.011 |
|  | [-0.039,0.10] | [-0.089,0.038] | [-0.065,0.072] | [-0.043,-0.0073] | [-0.0029,0.056] | [-0.049,0.026] |
| 2019🞨MC | Ref. | Ref. | Ref. | Ref. | Ref. | Ref. |
|  |  |  |  |  |  |  |
| 2020🞨MC | -0.0025 | -0.0049 | 0.02 | -0.022 | 0.013 | -0.0057 |
|  | [-0.060,0.055] | [-0.068,0.058] | [-0.043,0.082] | [-0.053,0.0081] | [-0.022,0.048] | [-0.049,0.037] |
| 2021🞨MC | 0.0086 | -0.0044 | 0.018 | -0.014 | 0.0091 | -0.013 |
|  | [-0.063,0.080] | [-0.046,0.038] | [-0.018,0.055] | [-0.033,0.0059] | [-0.029,0.047] | [-0.052,0.027] |
| 2022🞨MC | 0.032 | -0.0023 | 0.012 | -0.011 | 0.029* | -0.032 |
|  | [-0.022,0.086] | [-0.053,0.049] | [-0.038,0.061] | [-0.026,0.0051] | [0.0024,0.056] | [-0.067,0.0028] |
| 2023🞨MC | -0.014 | 0.024 | 0.043 | -0.026* | 0.017 | -0.015 |
|  | [-0.085,0.056] | [-0.026,0.074] | [-0.0018,0.088] | [-0.046,-0.0062] | [-0.012,0.046] | [-0.052,0.021] |
|  |  |  |  |  |  |  |
| *Dep. Mean in 2019* | 0.65 | 0.32 | 0.27 | 0.044 | 0.91 | 0.12 |
| *N* | 69168 | 69168 | 69111 | 68476 | 70167 | 69893 |
|  |  |  |  |  |  |  |
| ***Panel B: Medicaid Eligible plus 100% FPL Sample*** | | | | | | |
| 2016🞨MC | 0.037 | -0.035 | -0.012 | -0.033 | 0.011 | -0.0036 |
|  | [-0.0084,0.083] | [-0.12,0.046] | [-0.077,0.052] | [-0.072,0.0059] | [-0.019,0.041] | [-0.037,0.030] |
| 2017🞨MC | 0.077*** | -0.027 | -0.012 | -0.0095 | 0.017 | 0.0011 |
|  | [0.034,0.12] | [-0.068,0.014] | [-0.046,0.021] | [-0.031,0.012] | [-0.0072,0.041] | [-0.034,0.036] |
| 2018🞨MC | 0.073* | -0.057 | -0.016 | -0.038* | 0.019* | -0.0028 |
|  | [0.0029,0.14] | [-0.14,0.025] | [-0.077,0.045] | [-0.074,-0.0032] | [0.00050,0.038] | [-0.034,0.029] |
| 2019🞨MC | Ref. | Ref. | Ref. | Ref. | Ref. | Ref. |
|  |  |  |  |  |  |  |
| 2020🞨MC | 0.04 | -0.032 | -0.00073 | -0.037* | 0.017* | -0.012 |
|  | [-0.0067,0.087] | [-0.090,0.027] | [-0.052,0.050] | [-0.065,-0.0081] | [0.00018,0.033] | [-0.032,0.0091] |
| 2021🞨MC | 0.042 | -0.026 | -0.0047 | -0.021 | 0.0094 | -0.011 |
|  | [-0.0050,0.088] | [-0.064,0.012] | [-0.038,0.028] | [-0.054,0.013] | [-0.020,0.039] | [-0.043,0.021] |
| 2022🞨MC | 0.040* | -0.021 | 0.0025 | -0.026 | 0.0096 | -0.0039 |
|  | [0.0078,0.073] | [-0.072,0.030] | [-0.040,0.045] | [-0.066,0.014] | [-0.032,0.051] | [-0.052,0.044] |
| 2023🞨MC | 0.032 | -0.015 | -0.0013 | -0.029 | 0.0077 | -0.0014 |
|  | [-0.0080,0.073] | [-0.072,0.042] | [-0.044,0.041] | [-0.067,0.0092] | [-0.017,0.032] | [-0.029,0.027] |
|  |  |  |  |  |  |  |
| *Dep. Mean in 2019* | 0.53 | 0.45 | 0.38 | 0.065 | 0.92 | 0.11 |
| *N* | 131039 | 131039 | 130985 | 129052 | 132567 | 131996 |
| Notes: 95% confidence intervals are in brackets with state clustered standard errors. The estimates were obtained from a difference-in-difference event-study regression that estimates year-by-year differences in outcomes from 2016 to 2023 versus 2019 as reference year between the treatment and control groups. All models adjusted for age, sex, race/ethnicity, total number of children living in the household, highest educational level of parents/caregivers, marital status, any household employment, family income, Medicaid expansion status, state unemployment rate, survey year, and state of residence. Additionally, these models also control the interactions between state and income as well as interactions between year and income. All models are weighed by the NSCH sampling weights to yield nationally representative estimates.  *p<0.05, **p<0.01, ***p<0.001; Abbreviation: FFCRA, Families First Coronavirus Response Act; NSCH, National Survey of Children’s Health; MC, States without 12-month continuous Medicaid Coverage before the FFCRA in 2019 (i.e., the treatment group indicator). | | | | | | |

| Appendix Table 7. Difference-in-Differences Event Study Estimates of the Federal Medicaid Continuous Coverage Requirement Effects on Children’s Access to Care using Alternate Samples | | | | |
| --- | --- | --- | --- | --- |
|  | Usual place for sick care | Usual place for preventive care | Have a personal doctor | Unmet care needs |
| ***Panel A: Medicaid Eligible Sample*** | | | | |
| 2016🞨MC | -0.0068 | -0.0069 | -0.027 | -0.011 |
|  | [-0.049,0.036] | [-0.056,0.043] | [-0.078,0.024] | [-0.027,0.0043] |
| 2017🞨MC | -0.005 | 0.0036 | -0.0042 | -0.0019 |
|  | [-0.060,0.050] | [-0.054,0.061] | [-0.056,0.048] | [-0.027,0.023] |
| 2018🞨MC | -0.016 | 0.024 | 0.055 | -0.012 |
|  | [-0.051,0.019] | [-0.027,0.076] | [-0.012,0.12] | [-0.033,0.0088] |
| 2019🞨MC | Ref. | Ref. | Ref. | Ref. |
|  |  |  |  |  |
| 2020🞨MC | -0.045** | -0.017 | -0.019 | -0.0077 |
|  | [-0.075,-0.015] | [-0.064,0.029] | [-0.051,0.014] | [-0.033,0.018] |
| 2021🞨MC | 0.046 | 0.042 | 0.041 | -0.027** |
|  | [-0.0025,0.095] | [-0.019,0.10] | [-0.0019,0.084] | [-0.047,-0.0070] |
| 2022🞨MC | -0.0089 | 0.018 | 0.0083 | -0.027* |
|  | [-0.043,0.025] | [-0.015,0.050] | [-0.027,0.044] | [-0.053,-0.0013] |
| 2023🞨MC | 0.018 | 0.01 | 0.0076 | -0.01 |
|  | [-0.041,0.078] | [-0.036,0.057] | [-0.060,0.075] | [-0.034,0.014] |
|  |  |  |  |  |
| *Dep. Mean in 2019* | 0.7 | 0.87 | 0.62 | 0.035 |
| *N* | 70106 | 69883 | 70029 | 70130 |
|  |  |  |  |  |
| ***Panel B: Medicaid Eligible plus 100% FPL Sample*** | | | | |
| 2016🞨MC | 0.015 | -0.0023 | -0.021 | -0.0054 |
|  | [-0.017,0.046] | [-0.038,0.033] | [-0.064,0.022] | [-0.017,0.0067] |
| 2017🞨MC | 0.0048 | 0.0068 | -0.00079 | -0.0035 |
|  | [-0.031,0.040] | [-0.025,0.038] | [-0.043,0.041] | [-0.021,0.014] |
| 2018🞨MC | 0.01 | 0.027 | 0.037 | -0.012 |
|  | [-0.031,0.051] | [-0.018,0.072] | [-0.014,0.089] | [-0.025,0.0018] |
| 2019🞨MC | Ref. | Ref. | Ref. | Ref. |
|  |  |  |  |  |
| 2020🞨MC | -0.025* | -0.0099 | -0.0051 | -0.0079 |
|  | [-0.047,-0.0032] | [-0.050,0.030] | [-0.037,0.026] | [-0.024,0.0087] |
| 2021🞨MC | 0.035* | 0.037 | 0.027 | -0.017** |
|  | [0.0043,0.065] | [-0.00079,0.075] | [-0.015,0.070] | [-0.029,-0.0050] |
| 2022🞨MC | 0.0083 | 0.02 | 0.019 | -0.022** |
|  | [-0.018,0.035] | [-0.0060,0.046] | [-0.011,0.050] | [-0.038,-0.0063] |
| 2023🞨MC | 0.029 | 0.017 | 0.012 | -0.0027 |
|  | [-0.014,0.071] | [-0.019,0.054] | [-0.032,0.057] | [-0.018,0.013] |
|  |  |  |  |  |
| *Dep. Mean in 2019* | 0.73 | 0.89 | 0.66 | 0.035 |
| *N* | 132457 | 132066 | 132367 | 132541 |
| Notes: 95% confidence intervals are in brackets with state clustered standard errors. The estimates were obtained from a difference-in-difference event-study regression that estimates year-by-year differences in outcomes from 2016 to 2023 versus 2019 as reference year between the treatment and control groups. All models adjusted for age, sex, race/ethnicity, total number of children living in the household, highest educational level of parents/caregivers, marital status, any household employment, family income, Medicaid expansion status, state unemployment rate, survey year, and state of residence. Additionally, these models also control the interactions between state and income as well as interactions between year and income. All models are weighed by the NSCH sampling weights to yield nationally representative estimates.  *p<0.05, **p<0.01, ***p<0.001; Abbreviation: FFCRA, Families First Coronavirus Response Act; NSCH, National Survey of Children’s Health; MC, States without 12-month continuous Medicaid Coverage before the FFCRA in 2019 (i.e., the treatment group indicator). | | | | |

| Appendix Table 8. Difference-in-Differences Event Study Estimates of the Federal Medicaid Continuous Coverage Requirement Effects on Children’s Use of Health Servces by Type using Alternate Samples | | | | | | |
| --- | --- | --- | --- | --- | --- | --- |
|  | Any medical visits | Any preventive Check-up | Any mental visits | Any specialist visits | Any ED visits | Any hospital stay |
| ***Panel A: Medicaid Eligible Sample*** | | | | | | |
| 2016🞨MC | -0.011 | 0.0059 | -0.00078 | -0.02 | -0.0055 | N/A |
|  | [-0.075,0.053] | [-0.060,0.072] | [-0.026,0.025] | [-0.054,0.015] | [-0.045,0.034] |  |
| 2017🞨MC | -0.013 | -0.0029 | -0.014 | -0.054** | 0.02 | N/A |
|  | [-0.074,0.047] | [-0.059,0.053] | [-0.044,0.016] | [-0.094,-0.014] | [-0.038,0.077] |  |
| 2018🞨MC | 0.0037 | 0.018 | -0.0073 | -0.027 | 0.018 | -0.00075 |
|  | [-0.071,0.078] | [-0.050,0.086] | [-0.038,0.023] | [-0.063,0.0088] | [-0.028,0.064] | [-0.026,0.024] |
| 2019🞨MC | Ref. | Ref. | Ref. | Ref. | Ref. | Ref. |
|  |  |  |  |  |  |  |
| 2020🞨MC | 0.0026 | 0.036 | -0.016 | -0.017 | 0.0016 | -0.0049 |
|  | [-0.055,0.060] | [-0.036,0.11] | [-0.049,0.016] | [-0.064,0.030] | [-0.039,0.043] | [-0.025,0.015] |
| 2021🞨MC | 0.0099 | 0.03 | -0.018 | -0.016 | 0.033 | 0.0036 |
|  | [-0.037,0.056] | [-0.022,0.082] | [-0.048,0.013] | [-0.048,0.016] | [-0.019,0.084] | [-0.014,0.022] |
| 2022🞨MC | 0.029 | 0.029 | 0.0048 | -0.025 | 0.025 | 0.0044 |
|  | [-0.046,0.10] | [-0.034,0.092] | [-0.021,0.031] | [-0.062,0.012] | [-0.0065,0.056] | [-0.016,0.025] |
| 2023🞨MC | 0.016 | 0.026 | -0.01 | -0.034 | 0.015 | -0.0003 |
|  | [-0.038,0.070] | [-0.030,0.081] | [-0.043,0.023] | [-0.068,0.00065] | [-0.022,0.052] | [-0.020,0.019] |
|  |  |  |  |  |  |  |
| *Dep. Mean in 2019* | 70314 | 0.78 | 0.099 | 0.1 | 0.24 | 0.039 |
| *N* | 0.84 | 69880 | 70070 | 69941 | 69999 | 56056 |
|  |  |  |  |  |  |  |
| ***Panel B: Medicaid Eligible plus 100% FPL Sample*** | | | | | | |
| 2016🞨MC | -0.005 | 0.012 | -0.0021 | -0.025 | 0.0016 | N/A |
|  | [-0.057,0.047] | [-0.039,0.063] | [-0.023,0.018] | [-0.058,0.0075] | [-0.036,0.040] |  |
| 2017🞨MC | 0.0056 | 0.027 | -0.016 | -0.049** | 0.013 | N/A |
|  | [-0.036,0.047] | [-0.011,0.065] | [-0.042,0.0089] | [-0.079,-0.018] | [-0.034,0.060] |  |
| 2018🞨MC | 0.0089 | 0.026 | 0.00085 | -0.026 | 0.017 | 0.0083 |
|  | [-0.042,0.060] | [-0.018,0.070] | [-0.024,0.026] | [-0.054,0.0010] | [-0.022,0.056] | [-0.012,0.029] |
| 2019🞨MC | Ref. | Ref. | Ref. | Ref. | Ref. | Ref. |
|  |  |  |  |  |  |  |
| 2020🞨MC | [-0.036,0.072] | 0.043 | -0.018 | -0.019 | 0.005 | 0.0046 |
|  | 0.012 | [-0.017,0.10] | [-0.045,0.0091] | [-0.042,0.0040] | [-0.033,0.044] | [-0.013,0.022] |
| 2021🞨MC | [-0.024,0.048] | 0.03 | -0.021 | -0.019 | 0.022 | 0.0095 |
|  | 0.021 | [-0.016,0.077] | [-0.048,0.0065] | [-0.052,0.014] | [-0.020,0.065] | [-0.0044,0.023] |
| 2022🞨MC | [-0.041,0.084] | 0.03 | -0.0059 | -0.018 | 0.018 | 0.012 |
|  | 0.013 | [-0.020,0.079] | [-0.025,0.013] | [-0.048,0.012] | [-0.018,0.055] | [-0.0047,0.028] |
| 2023🞨MC | [-0.029,0.055] | 0.03 | -0.016 | -0.029* | 0.015 | 0.0066 |
|  | [-0.036,0.072] | [-0.0083,0.068] | [-0.039,0.0076] | [-0.056,-0.00086] | [-0.023,0.052] | [-0.013,0.026] |
|  |  |  |  |  |  |  |
| *Dep. Mean in 2019* | 132814 | 0.8 | 0.096 | 0.12 | 0.22 | 0.037 |
| *N* | 0.85 | 132072 | 132394 | 132173 | 132311 | 104654 |
|  |  |  |  |  |  |  |
| Notes: 95% confidence intervals are in brackets with state clustered standard errors. The estimates were obtained from a difference-in-difference event-study regression that estimates year-by-year differences in outcomes from 2016 to 2023 versus 2019 as reference year between the treatment and control groups. The 2016 and 2017 estimates for any hospital stay have been suppressed because no data are available for these years. All models adjusted for age, sex, race/ethnicity, total number of children living in the household, highest educational level of parents/caregivers, marital status, any household employment, family income, Medicaid expansion status, state unemployment rate, survey year, and state of residence. Additionally, these models also control the interactions between state and income as well as interactions between year and income. All models are weighed by the NSCH sampling weights to yield nationally representative estimates.  *p<0.05, **p<0.01, ***p<0.001; Abbreviation: FFCRA, Families First Coronavirus Response Act; NSCH, National Survey of Children’s Health; MC, States without 12-month continuous Medicaid Coverage before the FFCRA in 2019 (i.e., the treatment group indicator). | | | | | | |

| Appendix Table 9. Difference-in-Differences Event Study Estimates of the Federal Medicaid Continuous Coverage Requirement Effects on Children’s Health Status as Rated by Parents/Caregivers using Alternate Samples | | |
| --- | --- | --- |
|  | Excellent/very good health | Poor/fair health |
| ***Panel A: Medicaid Eligible Sample*** |  |  |
| 2016🞨MC | 0.019 | 0.0023 |
|  | [-0.014,0.052] | [-0.018,0.023] |
| 2017🞨MC | 0.054* | -0.000052 |
|  | [0.0079,0.100] | [-0.015,0.015] |
| 2018🞨MC | 0.044 | 0.0076 |
|  | [-0.0063,0.095] | [-0.017,0.033] |
| 2019🞨MC | Ref. | Ref. |
|  |  |  |
| 2020🞨MC | 0.051* | -0.0057 |
|  | [0.0079,0.095] | [-0.030,0.019] |
| 2021🞨MC | 0.053* | -0.002 |
|  | [0.00085,0.10] | [-0.034,0.030] |
| 2022🞨MC | 0.0024 | -0.007 |
|  | [-0.039,0.043] | [-0.023,0.0085] |
| 2023🞨MC | 0.041* | 0.00026 |
|  | [0.0040,0.079] | [-0.013,0.014] |
|  |  |  |
| *Dep. Mean in 2019* | 0.85 | 0.024 |
| *N* | 70244 | 70244 |
|  |  |  |
| ***Panel B: Medicaid Eligible plus 100% FPL Sample*** | |  |
| 2016🞨MC | 0.017 | -0.0011 |
|  | [-0.00044,0.034] | [-0.011,0.0093] |
| 2017🞨MC | 0.029 | -0.0013 |
|  | [-0.014,0.071] | [-0.011,0.0085] |
| 2018🞨MC | 0.021 | 0.0026 |
|  | [-0.0047,0.047] | [-0.012,0.018] |
| 2019🞨MC | Ref. | Ref. |
|  |  |  |
| 2020🞨MC | 0.028* | -0.0076 |
|  | [0.0036,0.052] | [-0.021,0.0056] |
| 2021🞨MC | 0.038* | -0.0037 |
|  | [0.0063,0.069] | [-0.022,0.015] |
| 2022🞨MC | 0.0033 | -0.0037 |
|  | [-0.018,0.025] | [-0.013,0.0052] |
| 2023🞨MC | 0.029** | -0.0015 |
|  | [0.0076,0.050] | [-0.0098,0.0068] |
|  |  |  |
| *Dep. Mean in 2019* | 0.87 | 0.021 |
| *N* | 132672 | 132672 |
| Notes: 95% confidence intervals are in brackets with state clustered standard errors. The estimates were obtained from a difference-in-difference event-study regression that estimates year-by-year differences in outcomes from 2016 to 2023 versus 2019 as reference year between the treatment and control groups. All models adjusted for age, sex, race/ethnicity, total number of children living in the household, highest educational level of parents/caregivers, marital status, any household employment, family income, Medicaid expansion status, state unemployment rate, survey year, and state of residence. Additionally, these models also control the interactions between state and income as well as interactions between year and income. All models are weighed by the NSCH sampling weights to yield nationally representative estimates.  *p<0.05, **p<0.01, ***p<0.001; Abbreviation: FFCRA, Families First Coronavirus Response Act; NSCH, National Survey of Children’s Health; MC, States without 12-month continuous Medicaid Coverage before the FFCRA in 2019 (i.e., the treatment group indicator). | | |
